# Mitonuclear incompatibility as a hidden driver behind the genome ancestry of African admixed cattle

**DOI:** 10.1186/s12915-021-01206-x

**Published:** 2022-01-17

**Authors:** Taehyung Kwon, Kwondo Kim, Kelsey Caetano-Anolles, Samsun Sung, Seoae Cho, Choongwon Jeong, Olivier Hanotte, Heebal Kim

**Affiliations:** 1 Department of Agricultural Biotechnology and Research Institute of Agriculture and Life Sciences, Seoul National University, Seoul, South Korea; 2 eGnome, Inc, Seoul, South Korea; 3 Callout Biotech, Albuquerque, New Mexico United States; 4 School of Biological Sciences, Seoul National University, Seoul, South Korea; 5 School of Life Sciences, University of Nottingham, Nottingham, UK; 6 LiveGene, International Livestock Research Institute (ILRI), Addis Ababa, Ethiopia; 7 The Centre for Tropical Livestock Genetics and Health (CTLGH), The Roslin Institute, The University of Edinburgh, Edinburgh, UK; 8 Interdisciplinary Program in Bioinformatics, Seoul National University, Seoul, South Korea

**Keywords:** African cattle, Mitonuclear incompatibility, Admixture, Phylogeny, Selection signatures, Approximate Bayesian computation

## Abstract

**Background:**

Africa is an important watershed in the genetic history of domestic cattle, as two lineages of modern cattle, *Bos taurus* and *B. indicus*, form distinct admixed cattle populations. Despite the predominant *B. indicus* nuclear ancestry of African admixed cattle, *B. indicus* mitochondria have not been found on the continent. This discrepancy between the mitochondrial and nuclear genomes has been previously hypothesized to be driven by male-biased introgression of Asian *B. indicus* into ancestral African *B. taurus*. Given that this hypothesis mandates extreme demographic assumptions relying on random genetic drift, we propose a novel hypothesis of selection induced by mitonuclear incompatibility and assess these hypotheses with regard to the current genomic status of African admixed cattle.

**Results:**

By analyzing 494 mitochondrial and 235 nuclear genome sequences, we first confirmed the genotype discrepancy between mitochondrial and nuclear genome in African admixed cattle: the absence of *B. indicus* mitochondria and the predominant *B. indicus* autosomal ancestry. We applied approximate Bayesian computation (ABC) to assess the posterior probabilities of two selection hypotheses given this observation. The results of ABC indicated that the model assuming both male-biased *B. indicus* introgression and selection induced by mitonuclear incompatibility explains the current genomic discrepancy most accurately. Subsequently, we identified selection signatures at autosomal loci interacting with mitochondria that are responsible for integrity of the cellular respiration system. By contrast with *B. indicus*-enriched genome ancestry of African admixed cattle, local ancestries at these selection signatures were enriched with *B. taurus* alleles, concurring with the key expectation of selection induced by mitonuclear incompatibility.

**Conclusions:**

Our findings support the current genome status of African admixed cattle as a potential outcome of male-biased *B. indicus* introgression, where mitonuclear incompatibility exerted selection pressure against *B. indicus* mitochondria. This study provides a novel perspective on African cattle demography and supports the role of mitonuclear incompatibility in the hybridization of mammalian species.

**Supplementary Information:**

The online version contains supplementary material available at 10.1186/s12915-021-01206-x.

## Background

Modern cattle populations can be divided mainly into two major lineages, humpless “taurine” (referring to *Bos taurus*) and humped “zebu” (referring to *Bos indicus*; also known as indicine). These two lineages stem from different subspecies of wild aurochs *Bos primigenius ssp* [1, 2] that diverged around 200,000 to less than 1 million years ago [1, 3–5]. Archaeological evidence suggests that the ancestors of taurine and zebu cattle arose from two different domestication events that occurred around 10,500 BP in the Fertile Crescent and 8000 BP in the Indus valley, respectively [1, 6].

Humpless taurine cattle were the first domesticated bovine species introduced into Africa. They arrived in northeastern Africa and subsequently spread over the continent [7]. Humped zebu cattle domesticated in South Asia were later introduced to Africa around AD 700, along with the Arab settlements and the development of Swahili civilizations on the eastern coasts of Africa [7, 8]. African-specific hybrid cattle populations have subsequently arisen from the admixture of these two ancestral cattle populations [8]. Recently, a major taurine × zebu cattle admixture event in the Horn of Africa was suggested to date back to 750–1050 years ago [9]. These admixed cattle have dispersed toward the west and south of the continent by continuous admixture with local African taurine populations [10]. The dispersal of the admixed cattle may have been accelerated during the rinderpest epidemics that occurred in the nineteenth and early twentieth century [7]. Today, indigenous African cattle populations are largely composed of African humpless taurine, African humped zebu, sanga (African humpless taurine × African humped zebu), and zenga (sanga × African humped zebu) [11]. According to the most recent study of African cattle classification [9], the term “African humped cattle” is used to consolidate the admixed cattle populations including African zebu, sanga, and zenga. In addition, Sheko population that was previously classified as African sanga [12] is now classified as a distinct subgroup “Sheko” of African humped cattle [9].

Phylogenetic studies based on microsatellites and single-nucleotide polymorphisms (SNPs) have supported taurine × zebu admixture across most cattle populations in the African continent [9, 13–15]. Moreover, microsatellite-based studies have reported the predominance of zebu *Y* chromosome throughout the continent [16–18]. In contrast, although supported by only a few African humped cattle samples, previous studies of mitochondrial phylogeny have reported the sole presence of taurine mitochondrial DNA lineages in African cattle: haplogroups T and Q [18–22]. A breeding scheme focusing on zebu male has been postulated to be at the root of this discrepancy between the mitochondrial and nuclear ancestries of the present-day African cattle [16–18, 23]. This hypothesis is based on (i) unbalanced sex ratio in the zebu herds migrated from South Asia, (ii) pastoralists’ preferences for male zebu phenotypes such as their large body size, and/or (iii) the male-biased breeding structure of African cattle herds, where typically only a few sires will contribute to the next generation [24–27]. Nevertheless, the genomic discrepancy in African humped cattle has not been empirically explained yet.

To explain this genomic discrepancy, we simulate a novel demographic scenario implementing a recently suggested hypothesis of “mitonuclear incompatibility” [28]. The mitonuclear incompatibility hypothesis is based on to the genetic incompatibility between mitochondria and nucleus that can affect fitness of the organism. It is often speculated to be an evolutionary driver maintaining the genomic integrity of a species, thus playing an important role in speciation or hybridization [28, 29]. The compact genome of the eukaryotic mitochondrion carries only a few core genes which conduct cellular respiration, a fundamental of organismal viability. As these mitochondrial genes must cooperate with many nuclear genes whose products function in mitochondria (N-mt genes), a mismatch between mitochondrial and N-mt genotypes may result in dysfunctional cellular respiration system [30, 31]. That is, hybrid mitochondria-nucleus pairs from different parental lineages may have impaired oxidative phosphorylation in the cells, which in turn may reduce the fitness of the hybrids (e.g., fecundity and/or survivability) [30, 32–34]. In such a scenario, hybrids with mitonuclear compatible pairs would be favored through selection over hybrids with mitonuclear incompatible pairs [35, 36], which leads to the displacement of one kind of N-mt alleles and mitochondria [37].

In this study, we analyzed 494 mitogenomes and a whole-genome set of 162 African and 73 Eurasian cattle to assess the evolutionary scenarios behind the genetic make-up of the current African humped cattle. In parallel to the hypothesis of male-biased zebu introgression, we hypothesized selection induced by mitonuclear genetic incompatibility (also referred to as “mitonuclear selection”). First, we illustrated the contrast between the taurine-enriched mitochondrial ancestry and the zebu-enriched nuclear ancestry in African humped cattle. Next, we applied a robust Bayesian inference method, approximate Bayesian computation (ABC) [38], to test the hypotheses and assess if any of them can explain the observed genomic discrepancy of African humped cattle. We then identified selection signatures on N-mt genes that concur with mitonuclear selection. Our results suggest that mitochondrial and N-mt alleles has co-evolved via mitonuclear selection favoring taurine alleles in African humped cattle. These results are consistent with the mitonuclear incompatibility hypothesis, genetic incompatibilities between mitochondrial and N-mt genes exerting post-zygotic selection pressure in hybrids.

## Results

### Sequence data preparation

In this study, we analyzed whole-genome sequencing (WGS) data of 235 samples and 494 cattle mitochondrial genome sequences (Additional file 2: Table S2). African cattle samples are primarily classified into two major groups based on their morphologies [9, 12, 14, 15, 26, 39]: African humpless taurine cattle and African humped cattle. African humpless taurine group (AFT) refers to African cattle with *B. taurus* morphology (Domiaty, N’Dama, and Menofi breeds). African humped cattle group is further divided into African humped zebu (AFZ), African humped hybrids (HYB), and African humped Sheko (SHE). AFZ refers to African cattle with *B. indicus* morphology (Arsi, Barka, Butana, Ethiopian Boran, Goffa, Kenana, Kenyan Boran, Mursi, and Ogaden breeds). HYB includes sanga and zenga hybrids that have a distinct *B. taurus* × *B. indicus* hybrid morphology (Afar, Ankole, Fogera, Horro, Nguni, and Abigar breeds). SHE only includes the Sheko breed [9]. Eurasian cattle are also divided into two groups: European and Asian taurine (EUT/AST) and Asian zebu (ASZ).

Raw sequencing data of 235 WGS samples, which includes 162 African cattle and 73 Eurasian cattle, was retrieved from the NCBI Sequence Read Archive (SRA) database. The African cattle WGS data is composed of AFZ (9 breeds, *n* = 101), AFT (single breed, *n* = 13), HYB (4 breeds, *n* = 39), and SHE (single breed, *n* = 9). The Eurasian cattle WGS data is composed of AST (single breed, *n* = 23), EUT (3 breed, *n* = 30), and ASZ (3 breeds, *n* = 20). NCBI Biosample accession, morphological classification, and breed information for each sample is summarized in Additional file 1: Table S1. We mapped WGS reads against the autosomes of *B. taurus* reference UMD 3.1, which resulted in an average alignment rate of 98.56% and coverage of 98.44%. Mapping statistics for WGS data are summarized in Additional file 1: Table S1. Multi-sample variant calling resulted in 41,520,225 autosomal SNPs with an average genotyping rate of 0.9881.

For *Y* chromosome variant calling, we first imputed the sex information for each WGS sample based on the depth coverage of reads (mapping quality > 0) by mapping them against Btau 4.6 *Y* chromosome following the 1000 bull genome project pipeline [40]. The average depths of *X* and *Y* sex chromosomes were respectively divided by that of the autosome to obtain the depth ratios of sex chromosomes. From the depth ratios of *X* and *Y* chromosomes, 67 samples were imputed as male (20 AFZ, 7 HYB, 9 EUT, 12 AST, and 19 ASZ samples). Sex chromosome mapping statistics and imputed sex information is summarized in Additional file 1: Table S1. We obtained a total of 31,269 *Y* chromosomal SNPs from 67 male samples.

We generated mitochondrial sequences based on SNPs and indels called from WGS reads by mapping them against the *B. taurus* mitochondrial reference genome NC_006853 (T_AST_053RS). However, we failed to generate mitochondrial sequences for 19 of the 20 ASZ samples due to poor depth coverage of mitochondrial sequences, leaving one single Nelore mitochondrial genome (I_ASZ_001@). Detailed information of mitochondrial sequence assembly is summarized in Additional file 1: Table S1. We included 276 publicly available mitochondrial genomes of modern cattle, ancient cattle (auroch, *B. primigenius*), and outgroup taxa (yak, *B. grunniens*; bison, *Bison bison*). As a result, we aligned and trimmed 494 whole mitochondrial sequences of which morphological classification and breed information is summarized in Additional file 2: Table S2.

### Mitochondrial phylogenetic analyses

**Fig. 1:**
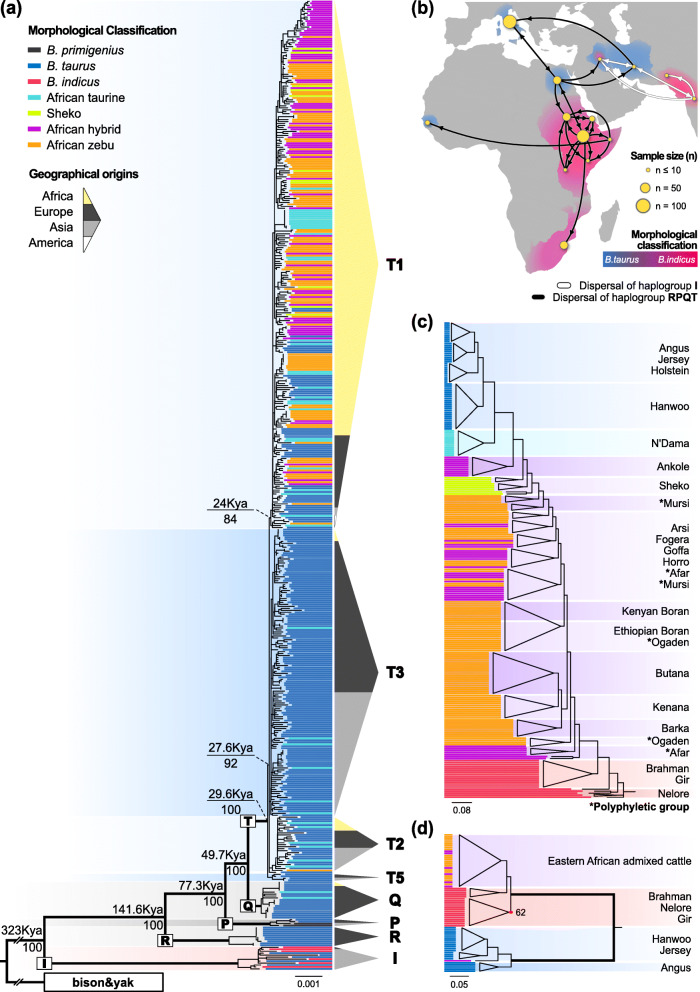
Phylogenetic signals based on mitochondrial, nuclear, and *Y* chromosomal sequences. **A** Maximum-likelihood tree using 494 mitochondrial genomes. Mean divergence time and ultrafast bootstrap value are shown for the nodes of haplogroup divergence. Composition of geographical origins is summarized in triangle charts for each haplogroup. **B** Phylogeography inferred using mitochondrial genomes of the samples found in Africa and the adjacent regions. Phylogeographic dispersals with Bayesian posterior probability less than 0.8 are omitted. **C**, **D** Maximum-likelihood tree using (**C**) autosomal SNPs and (**D**) *Y* chromosomal SNPs. Ultrafast bootstrap values over 0.8 are omitted. Nodes including three or more leaves of similar branch lengths are collapsed. **A**, **C**, **D** Each colored bar in phylogenetic trees indicates the morphological classification of each sample

Results of recent phylogenetic studies on the mitochondrial genome of cattle have indicated that the matrilineal lineages of *B. taurus* are represented by haplogroups T (with sub-haplogroups T, T1, T2, and T3), Q, P, and R, while those of *B. indicus* are represented by haplogroup I [4, 20, 21, 41–45]. Our maximum-likelihood (ML) phylogenies based on 494 mitochondrial genome sequences mainly agree with the previous phylogenetic classification (Additional file 2: Table S2), except for the placement of T_AFT_025 in the T3 sub-haplogroup which was previously reported to be placed in the T1 sub-haplogroup [20] (Fig. 1a). Regardless of their morphological classifications, all African cattle (*n* = 236) were assigned to taurine mitochondrial haplogroups Q (*n* = 2) and T (*n* = 234). African taurine cattle (*n* = 44) predominantly clustered in haplogroup T, more specifically T1 (32 non-Egyptian African taurine), T2 (6 Egyptian taurine), and T3 (6 Egyptian taurine), except for two Egyptian taurine belonging to haplogroup Q. Meanwhile, 180 of the 181 African humped cattle (AFZ, HYB, and SHE) were assigned to the T1 subgroup, with the exception of one Arsi individual belonging to sub-haplogroup T2. These results show a stark contrast to the zebu-like morphology of African humped cattle. This result provides the most substantial evidence so far of the fixation of taurine mitochondrial haplotype in African humped cattle, considering that only a small number of mitochondrial genomes of African humped cattle were available [20, 21, 46]. Interestingly, six Asian taurine cattle from Mongolia, China, Iran, and Iraq were included in haplogroup I, which exemplifies introgression of zebu mitochondria into taurine populations around South and East Asia.

Our Bayesian inference of evolutionary features [47] estimated the mean substitution rates for the *D*-loop, protein-coding genes, and non-coding tRNA/rRNA partitioned regions as 2.51 × 10^−7^, 2.50 × 10^−8^, and 1.37 × 10^−7^ substitutions per site per year, respectively. The estimated divergence time between taurine (RPQT) and zebu (I) agrees with previous reports (mean divergence time = 323 Kya; highest posterior density 95% interval = 219.2 ~ 423.6 Kya). The emergence of the most recent common ancestor for T haplogroup (mean divergence time = 29.6 Kya; highest posterior density 95% interval = 20.0 ~ 39.9 Kya) predated the previously reported times of ~ 16 Kya [4, 45, 48]. A low level of genetic variation within haplogroup T presumably resulted from a small effective sample size (ESS < 20) of the Bayesian inference of the tree-likelihood for T sub-haplogroups. Inter-haplogroup tree topologies concurred with those of ML tree, and intra-haplogroup tree topologies of Bayesian inference tree were omitted due to low posterior probabilities. To avoid the caveat of low variation while inferring the discrete phylogeography of the African population, we focused on populations from the region within and adjacent to Africa. Our phylogeography analysis was performed using 353 mtDNA sequences originated from Africa, western/southern Asia, and southern Europe (Fig. 1b). Phylogeographic inference results suggest two major dispersals of mitochondrial haplotypes, one for haplogroup I and the other for haplogroups RPQT. While haplogroup I was found only within southern and western Asia, taurine mito-lineages appeared to have spread into Europe, Asia, and Africa. The phylogeographic resolution of the Fertile Crescent population remained obscure, possibly due to a lack of sample size and a single geographical origin of the western Asian samples. The Egyptian population appeared to be the only African mitochondrial pool connected to the Eurasian mitochondrial pool, which contains 17 haplotypes from T1, six haplotypes from T2, six haplotypes from T3, and two haplotypes from Q. These results support the role of northeastern Africa as a channel of cattle migration between Africa and Eurasia and as the cradle of ancestral African cattle mtDNA. The majority of non-Egyptian African cattle mtDNA were assigned to T1 (204 out of 205), suggesting an early dispersal of Egyptian T1 founders throughout Africa. In contrast, T2 and T3 haplotypes were predominantly found in Asian cattle, with only four of 94 Asian haplotypes (one Iraqi and three Korean cattle) belonging to T1. This result indicates the difference of composition between the Asian mtDNA pool and the African mtDNA pool.

### Nuclear genome analyses

In contrast to the mitochondrial phylogeny, ML phylogenies based on autosomal and *Y* chromosomal SNPs indicate that the nuclear genomes of our eastern African humped cattle samples are predominantly introgressed with Asian zebu ancestry. African humped cattle were phylogenetically closer to ASZ than to EUT/AST or N’Dama (AFT), except for Ankole being closer to N’Dama (Fig. 1c). This phylogenetic clustering concurs with the phylogeny based on coding-region variants (Additional file 3: Fig. S1). The *Y* chromosomal phylogeny (Fig. 1d) showed a definite separation between Asian zebu *Y* (previously known as Y3) and taurine *Y* haplogroups (Y1 and Y2) [49], which concurs with the result of principal component analysis (PCA) (Additional file 3: Fig. S2). All African humped cattle except for one Ankole sample have *Y* chromosome of zebu origin, as in agreement with the previous reports [16, 18]. A single Ankole sample carrying taurine *Y* haplotype (SAMN04545542) was not embedded within EUT or AST taurine *Y* groups, supporting the presence of African-specific taurine *Y* haplogroup [50].

**Fig. 2:**
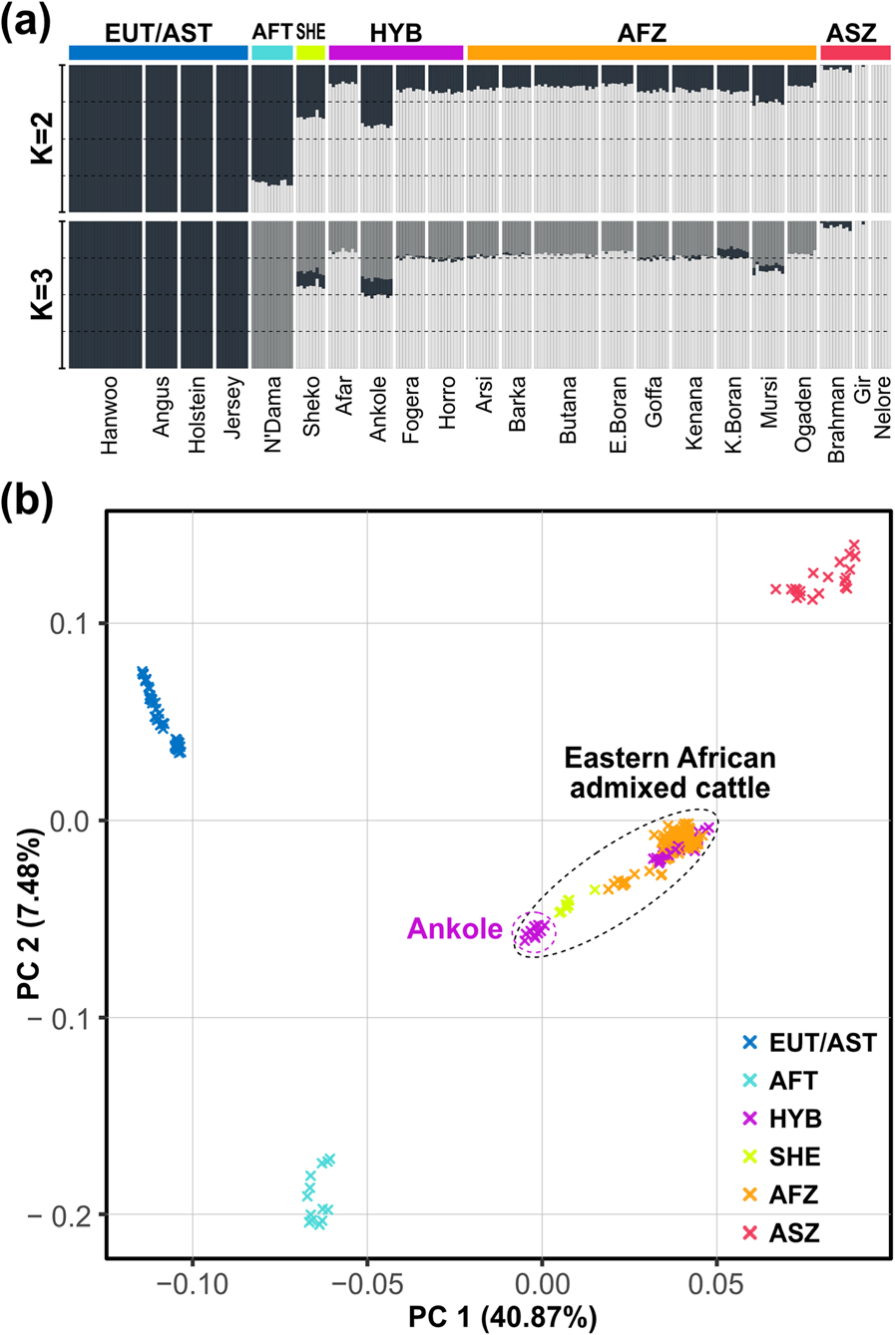
Population structures of African cattle. **A** Estimated genome-wide ancestries and **B** PCA plot based on autosomal SNPs. *K* indicates the number of ancestry components assumed in the ADMIXTURE analysis

The results of ML genome ancestry estimation and PCA using the autosomal variants agree with previous reports of zebu-enriched genome admixture in African humped cattle [9, 13–15]. Assuming two ancestry components (*K* = 2, Fig. 2a), unsupervised genetic clustering assigned one ancestry component to EUT/AST and the other to ASZ. The average proportion of the major ancestry of AFZ shared with ASZ was 83.5%, with the standard deviation of 3.36%. However, when we assume three ancestry components (*K* = 3, Fig. 2a), the ancestry component prevalently shared by EUT and AST was distinct from the one assigned to AFT. The ancestry component assigned to AFT took up the proportion of the zebu component shared by African humped cattle and ASZ, which suggests that the ancestry of N’Dama might be correlated with the zebu ancestry in African humped cattle. Overall, all African humped cattle shared more than 50% of zebu ancestry at *K* = 3, with an average of 75.5% ± 3.63% for AFZ, 77.6% ± 11.2% for HYB, and 55.9% ± 1.99% for SHE. PCA also supported this zebu-enriched admixture in African humped cattle by PC1 explaining 41% of the variance. SHE and Ankole were positioned toward AFT, separately for other African humped cattle populations (Fig. 2b). In general, the results of all genome-wide analyses illustrate the strong zebu influence in the admixed genome of eastern African humped cattle, which sharply contrasts with the result of the mitochondrial analyses presented above.

### Approximated Bayesian computation of selection hypotheses

Genomic analysis results indicate the discrepancy between the absence of zebu mitochondrial ancestries and the zebu-enriched autosomal and *Y* ancestries in African humped cattle. We tested two selection pressures behind this observation: (i) a previously suggested hypothesis of male zebu biased introgression “male-biased zebu selection” and (ii) a new hypothesis of selection induced by mitonuclear incompatibility “mitonuclear selection.” In order to assess these evolutionary forces given the genomic discrepancy, we performed a set of forward simulations under different demographic scenarios of ancestral population and selection models. We used in-house R scripts *abc_simulate.R* to run forward simulation and *abc_summarize.R* to perform ABC (https://github.com/TaehyungKwon/abc_simulation).

To apply realistic simulation hyperparameters, we first estimated the time of admixture and the change of the effective population size (*N*_*e*_) from autosomal variants of 101 AFZ samples. AFZ here accounts for the largest homogeneous population data of African humped cattle. As a result, we applied the time of the admixture at 110 generations ago (± 4.47) estimated with a single-pulse admixture model in ALDER [51]. Subsequently, *N*_*e*_ changes from the time of admixture to the present days were applied in simulation after estimation using SMC++ [52] (Additional file 3: Fig. S3). With these hyperparameters implemented, we then designed a pulse-like admixture model that Asian zebu immigrant population “pre-Z” and ancestral African taurine population “pre-T” are admixed into a single homogeneous population “post-A”.

**Fig. 3:**
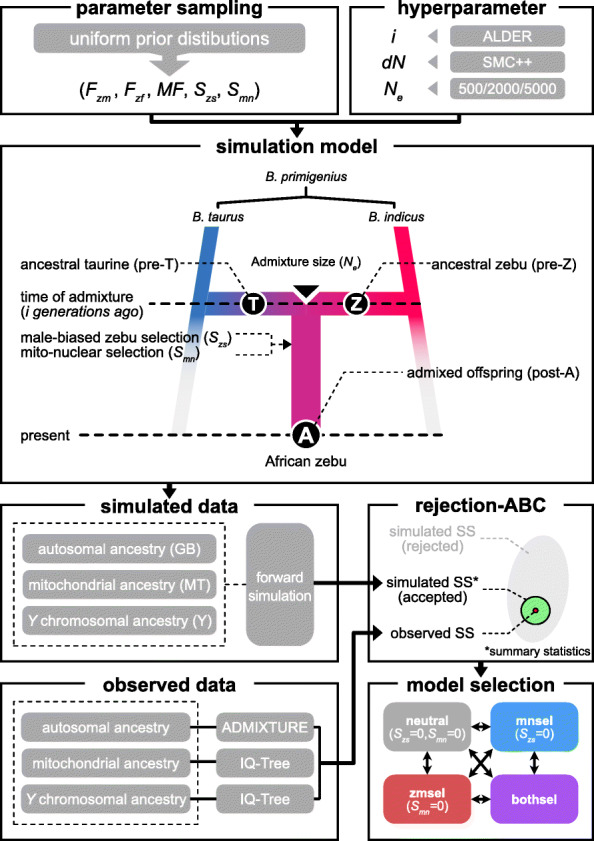
Scheme of the approximated Bayesian computation. The diagram describes (i) components of simulation, (ii) detailed process using the observed and simulated data, and (iii) model selection based on Bayes factor analysis

Summary statistics used in this simulation are the autosomal genetic background “GB,” mitochondrial haplotype “MT,” and *Y* chromosomal haplotype “Y” (Fig. 3). In addition, we designed haplotype at N-mt genes “MN” to implement mitonuclear selection. These summary statistics are distinguishable between zebu (e.g., GB = 1) and taurine (e.g., GB = 0). After simulation, we applied rejection ABC to compare GB, MT, and Y of simulated populations with the observed summary statistics estimated from 101 AFZ samples: zebu autosomal ancestry = 0.755, frequency of zebu mitochondrial haplotype = 0, and frequency of zebu *Y* chromosomal haplotype = 1.

Three demographic factors were parametrized; the proportion of zebu individuals in the male pool “*F*_*zm*_” randomly sampled from the prior distribution following uniform distribution *U*(0, 1), the proportion of zebu individuals in the female pool “*F*_*zf*_” from *U*(0, 0.5), and the male frequency within the population “*MF*” from *U*(0.05, 0.5) (Table 1). Two selection pressures were parametrized and randomly sampled for male-biased zebu selection “*S*_*zs*_” from *U*(0, 100) and mitonuclear selection “*S*_*mn*_” from *U*(0, 0.2) (Table 1). To test two selection hypotheses that are not exclusive, we generated four simulation models: (i) “neutral,” a model without any selection pressures with *S*_*mn*_ fixed to 0 and *S*_*zs*_ fixed to 0, (ii) “mnsel,” a model only assuming the mitonuclear selection with *S*_*zs*_ fixed to 0, (iii) “zmsel,” a model only assuming the male-biased zebu selection with *S*_*mn*_ fixed to 0, and (iv) “bothsel,” a model assuming both selection pressures. All models were simulated for 1 × 10^7^ replicates. For each simulation replicate, we obtained mean GB, mean MT, mean Y, and mean MN of the simulated population to compare with the observed summary statistics. With standardized Euclidean distances between the simulated and the observed summary statistics, we performed rejection ABC with tolerance of 0.01 and 0.001, respectively; for example, tolerance of 0.001 accepts simulated sets with the smallest distances of 0.1% [38, 53].

**Table 1:** Descriptions and prior distributions of the parameters used in the simulation model

Parameter	Description	Prior distribution
*F*_*zm*_	Proportion of zebu individuals in male population	*U*(0, 1)
*F*_*zf*_	Proportion of zebu individuals in female population	*U*(0, 0.5)
*MF*	Male frequency of the population	*U*(0.05, 0.5)
*S*_*zs*_	Selection coefficient for mitonuclear selection	*U*(0, 0.2)
*S*_*mn*_	Selection coefficient for male-biased zebu selection	*U*(0, 100)

To test the effect of genetic drift by increment of admixture size, we refer to the previous reports of *N*_*e*_ using WGS data of African cattle, which suggest *N*_*e*_ of African humped cattle breeds from 2000 to 10,000 at 110 generations ago [14, 54]. Accordingly, we set three *N*_*e*_ presets at 110 generation ago: 500, 2000, and 5000. *N*_*e*_ of 500 represents a small admixture size, assuming *N*_*e*_ of single unadmixed African cattle breed such as N’Dama [14]. *N*_*e*_ of 2000 and 5000 respectively represent approximates of single-population and multi-population *N*_*e*_ of African humped cattle. To determine if models are distinguishable, we compared the recall (the fraction of true positives among all predictions) of cross-validation by each level of *N*_*e*_ and tolerance (Additional file 3: Fig. S4). As a result, model distinguishability was the lowest with *N*_*e*_ of 500, while *N*_*e*_ of 2000 and 5000 showed similar recalls.

**Fig. 4:**
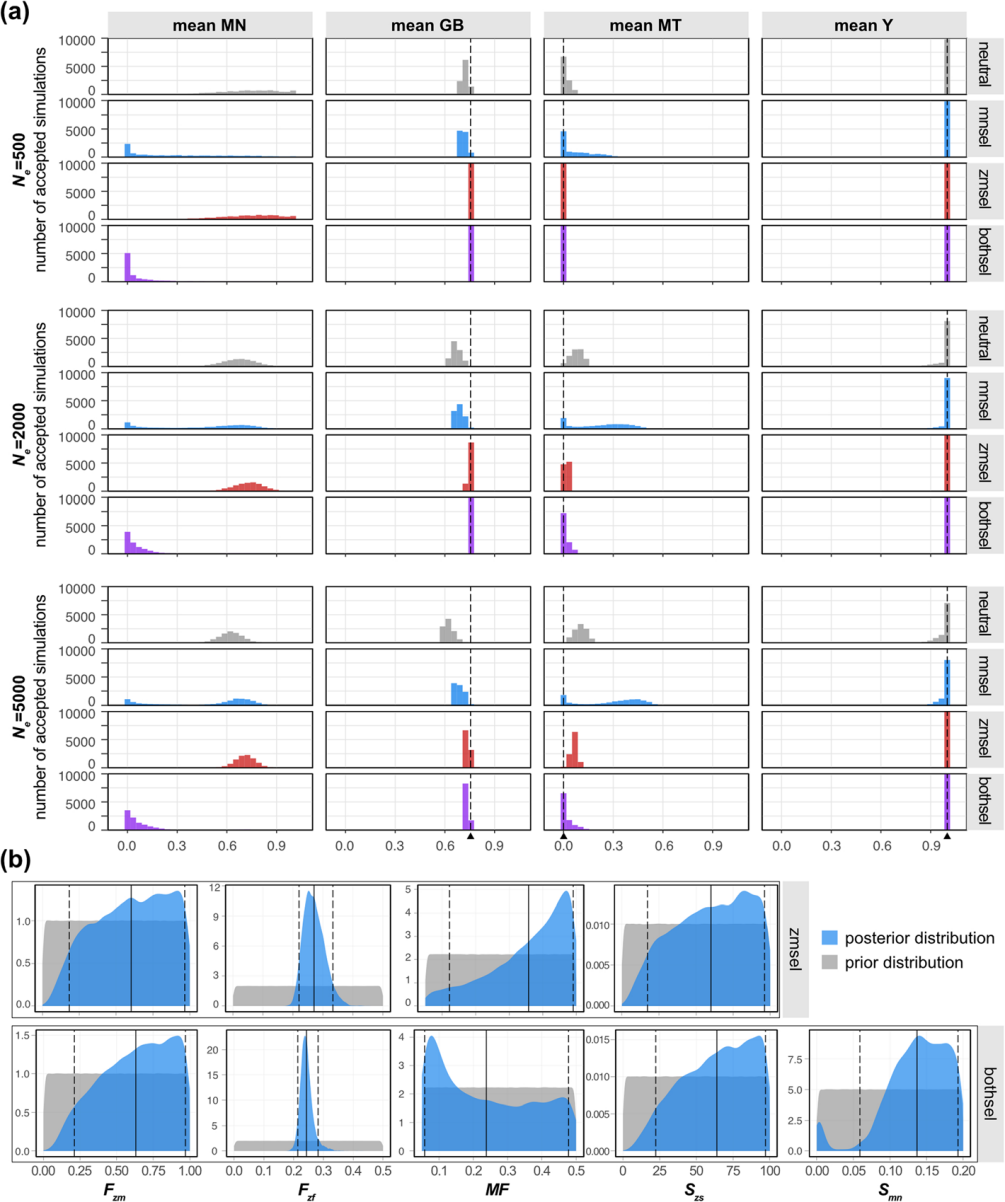
Results of the approximate Bayesian computation. **A** Distribution of the accepted summary statistics with tolerance of 0.001 (10,000 simulation replicates). Each column illustrates histogram of the mean values of each summary statistics from accepted simulations. Black dashed line indicates the observed value of each summary statistics. **B** Prior and posterior distributions of the parameters in “zmsel” and “bothsel” models with *N*_*e*_ of 5000 and tolerance of 0.001. Black solid line indicates mean of posterior distribution. Black dashed lines indicate 90% highest posterior density interval of the posterior distribution

We performed model selections by comparisons of the posterior probabilities between models, also known as Bayes factor analysis (Table 2). When *N*_*e*_ is set to 2000 and 5000, Bayes factor of “bothsel” given other models indicates that “bothsel” has substantial (Bayes factor ≥ 3) or very strong (Bayes factor ≥ 30) evidence over the second most probable model “zmsel” [55]*.* However, when *N*_*e*_ is set to 500, “zmsel” has higher posterior probability, without any evident posterior support (1/3 ≤ Bayes factor < 1) [55]. This trend also corresponds to the distances between the observed and accepted summary statistics, supporting “bothsel” over all other models when *N*_*e*_ is set to 2000 and 5000 (Additional file 3: Fig. 5). Both “zmsel” and “bothsel” with *N*_*e*_ of 500 showed almost zero of mean distances (0.004 ± 0.002 and 0.007 ± 0.004, respectively) between the accepted and observed summary statics. Accordingly, accepted simulations with tolerance of 0.001 under “zmsel” and “bothsel” converged to the observed summary statistics with all *N*_*e*_ (Fig. 4a). Goodness-of-fit tests revealed that the goodness-of-fit statistics between the observed data was placed inside of the null distributions “zmsel” and “bothsel”, while goodness-of-fit tests for “neutral” and “mnsel” models were rejected (*P* value < 0.05) in most cases (Additional file 3: Fig. S6). These results support that the models assuming male-biased zebu selection (“zmsel” and “bothsel”) likely reproduce the observed data.

**Table 2:** Results of Bayes factor analyses between simulation models. Bayes factor for M /M indicates posterior probability of model X divided by posterior probability of model Y. Bayes factor of 3 or more indicates support of model X over model Y, and Bayes factor of less than 1/3 indicates support of model Y over model X, as suggested by Jeffreys H [55].

***N***_***e***_	Tolerance	Bayes factor (***M***_**bothsel**_/***M***_**neutral**_)	Bayes factor (***M***_**bothsel**_/***M***_**mnsel**_)	Bayes factor (***M***_**bothsel**_/***M***_**zmsel**_)
500	0.01	5.28 × 10^2^	1.30 × 10^2^	6.31 × 10^−1^
500	0.001	8.17 × 10^2^	1.65 × 10^2^	5.84 × 10^−1^
2000	0.01	1.48 × 10^4^	5.24 × 10^3^	4.33 × 10^0^
2000	0.001	Larger than 10^309^	6.28 × 10^3^	3.67 × 10^0^
5000	0.01	3.92 × 10^5^	Larger than 10^309^	5.11 × 10^1^
5000	0.001	Larger than 10^309^	Larger than 10^309^	1.60 × 10^2^

Bayes factor for *M*_X_/*M*_Y_ [55].

Interestingly, when *N*_*e*_ is set to 500, accepted simulations under “neutral” seemingly converged to the observed summary statistics (Fig. 4a), which is supported by the mean distance under “neutral” model with *N*_*e*_ of 500 (0.213 ± 0.075) being similar to the mean distance under “bothsel” with *N*_*e*_ of 5000 (0.18 ± 0.039) (Additional file 3: Fig. S5). That is, in a small-sized admixture, the model assuming neutrality can reproduce the observed data as well, since the convergence in this demographic scenario heavily relies on random genetic drift. For realistic admixture sizes (*N*_*e*_ of 2000 and 5000), “neutral” model failed to reproduce the observed summary statistics, whereas only the models assuming male-biased zebu selection successfully reproduced them (Fig. 4a). As we increased *N*_*e*_ from 2000 to 5000, “zmsel” failed to reproduce the observed MT while “bothsel” reproducing both the observed MT and the observed GB (Fig. 4a), also shown in the result of the Bayes factor analysis (Table 2). In all four models, the demographic parameters accepted given the observed data reproduced predominance of zebu *Y* haplotype in any admixture size (Fig. 4a). These results support that when assuming a sizeable *N*_*e*_, the model assuming cooperation of both selection pressures elevates the reproducibility of the observed genome discrepancy in African humped cattle.

We here note that the model assuming only mitonuclear selection pressure, “mnsel,” does not favor the loss of zebu mitochondria without male-biased zebu selection (Fig. 4a). This result indicates the frequency-dependent nature of mitonuclear selection, supported by the difference of accepted MN between “mnsel” and “bothsel” (Fig. 4a). Unlike “mnsel,” accepted MNs of “bothsel” were close to zero that indicates taurine ancestries at N-mt loci (Fig. 4a), which suggests that mitonuclear selection would favor hybrids with the taurine MN and MT haplotypes only together with male-biased zebu selection (Fig. 4a).

Under “zmsel” model with tolerance of 0.001, the mean of posterior distribution of *F*_*zf*_ were 0.27~0.316 in all *N*_*e*_ presets (Additional file 3: Table S3). That is, although the presence of zebu female in population would hinder “zmsel” from achieving the observed MT, “zmsel” requires a substantial size of zebu female population to achieve the observed GB. Therefore, the initial involvement of zebu female in “zmsel” was not erased by random genetic drift when assuming *N*_*e*_ of 5000 (Fig. 4b), resulting in a failure of convergence of MT to the observed value (Fig. 4a). Moreover, the posterior distribution with tolerance 0.001 and *N*_*e*_ of 5000 suggests that posterior *MF* of “bothsel” is toward the lower end of the prior (0.238 ~ 0.278), whereas posterior *MF* of “zmsel” is toward the higher end of the prior (0.315~0.358) (Fig. 4b and Additional file 3: Table S3). Higher mean posterior *MF* value of “zmsel” compared to that of “bothsel” suggests that high *MF* can compensate for low *F*_*zf*_ to achieve zebu-enriched GB, thus aggravating random loss of zebu mitochondrial haplotype in “zmsel.” These results in posterior *F*_*zf*_ and *MF* suggest that the random loss of mitochondria works as a constraint for “zmsel” to incur lower posterior probability of the model than that of “bothsel.” To summarize, the model with both selections explains the current African humped cattle the best when assuming a realistic admixture size, as being able to induce the complete loss of a mitochondrial haplotype despite substantial zebu nuclear ancestry.

### Detection of mitonuclear selection signatures

**Fig. 5:**
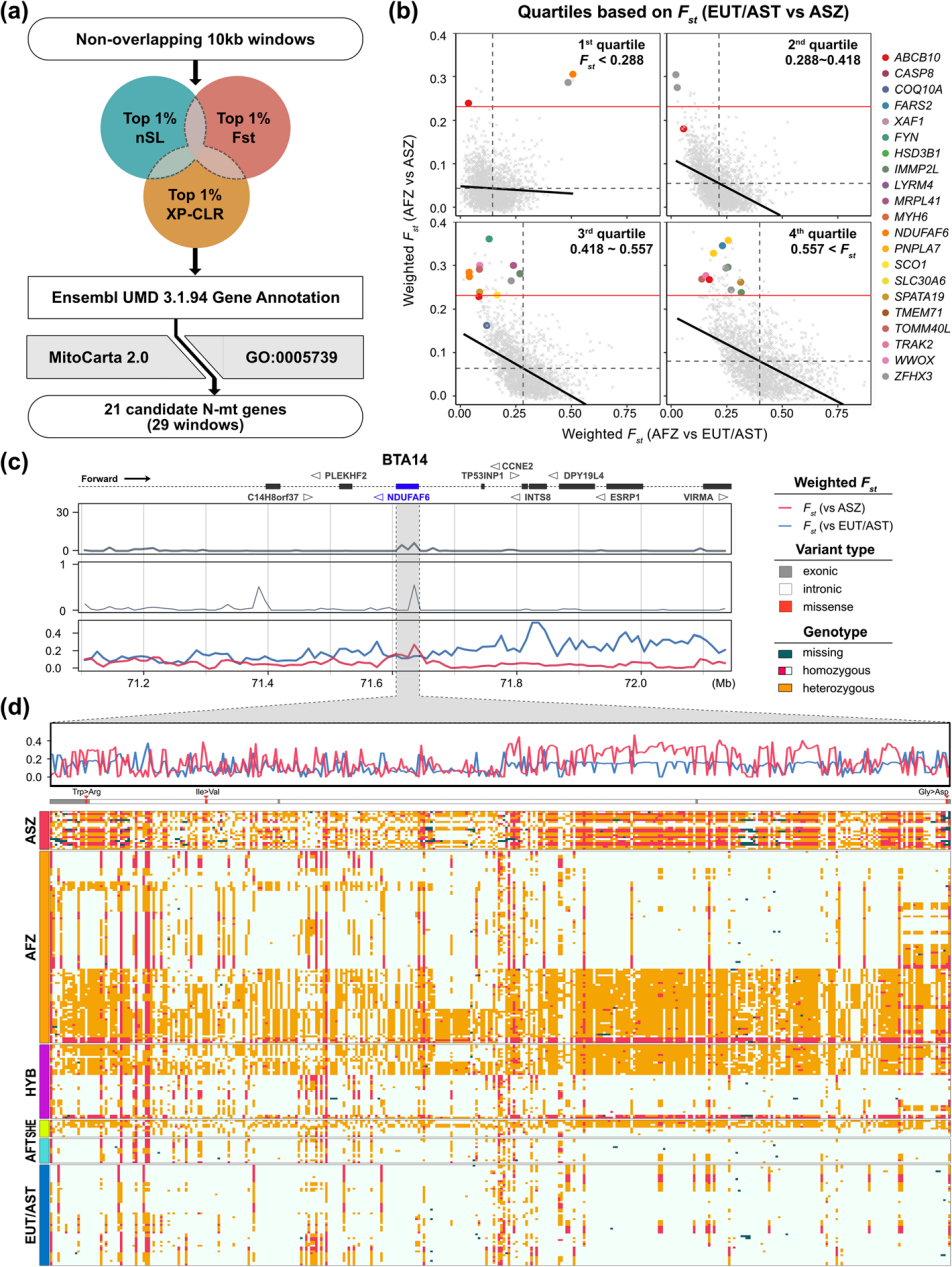
Summary of selection scan for mitonuclear selection signatures. **A** The diagram of selection scan process to detect mitonuclear selection signatures. **B** Population differentiation (*F*_*st*_) between AFZ and ASZ or between AFZ and EUT/AST for each 10 kb window overlapping with N-mt genes. Each window is stratified by *F*_*st*_ quartiles divided based on *F*_*st*_ between EUT/AST and ASZ. The black dashed line indicates the mean *F*_*st*_ of each axis at each quartile. The black solid line indicates the linear regression. The red solid line indicates the threshold of the top 1% cutoff of *F*_*st*_ between AFZ and ASZ. **C**, **D** Selection signatures and genotypes in a selection candidate *NDUFAF6* region. **C** Normalized XP-CLR (top), normalized nSL (middle), and weighted *F*_*st*_ (bottom) calculated for 10 kb non-overlapping windows in 1 Mb region adjacent to the candidate gene. **D** Weighted *F*_*st*_, variant types, and genotypes at SNP loci is visualized for the candidate gene region. Missense variants and corresponding amino acid substitutions are marked with red arrows. SNP loci with minor allele frequency of 0.05 or lower are omitted. Individuals within each population are hierarchically clustered based on genotypes

If mitonuclear incompatibility indeed played a role in shaping the genome of African cattle, we might expect to find signatures of selection in autosomal regions interacting with the mitochondria. In order to identify these selection signatures, we performed a combined selection scan on non-overlapping 10-kb windows using three different methods: Weir and Cockerham’s *F*_*st*_ [56], XP-CLR [57], and nSL [58] (Fig. 5a). We used ASZ as the reference zebu group in the cross-population selection analysis. The cutoff values for the top 1% of the three selection scans were 0.231 for the weighted *F*_*st*_, 3.805 for the normalized XP-CLR, and 0.621 for the normalized nSL. The top 1% intersection of the three scan results includes 310 10-kb windows overlapping with 205 candidate genes. Of these 310 windows, 234 were shared between *F*_*st*_ and XP-CLR, 32 between *F*_*st*_ and nSL, 23 between the XP-CLR and nSL, and 21 were common to the results of the three selection scans. It should be noted that the result of nSL showed the lowest correlations with those of *F*_*st*_ and XP-CLR (Pearson’s correlation coefficient of 0.0357 and 0.101; Additional file 3: Fig. S7).

To identify putative N-mt genes, we used a merged list of 1801 Ensembl UMD 3.194 [59] genes obtained from 1576 Human MitoCarta 2.0 one-to-one orthologous genes [60] and 1178 genes assigned to the GO:0005739~mitochondrion. A total of 21 N-mt genes were found within candidate windows, overlapping with 29 windows (22 windows in common between the *F*_*st*_ and the XP-CLR analyses, four windows in common between the *F*_*st*_ and nSL analyses, and three common windows between the XP-CLR and nSL analyses). The results of the selection scan of these 21 mitonuclear candidate genes are summarized in Table 3.

**Table 3:** Selection scan results for 21 candidate genes

Gene symbol	Window (chr:start-end)	normalized XP-CLR	normalized nSL	weighted ***F***_***st***_	Functional description
*LYRM4*	23:49320001-49330000	9.29	0.64	0.18	To bind cysteine desulfurase and help free inorganic sulfur for Fe/S clusters
*COQ10A*	5:57390001-57400000	6.31	0	0.31	Required for the function of coenzyme Q in the respiratory chain
*TOMM40L*	3:8290001-8300000	8.26	0	0.29	To participate in the import of precursors into mitochondria as potential channel-forming protein
*FARS2*	23:49010001-49020000	5.54	0.65	0.16	To transfer phenylalanine to tRNA and to participate in mitochondria for mitochondrial protein translation
*MRPL41*	11:105590001-105600000	8.98	0	0.35	To participate in protein synthesis within the mitochondrion as ribosomal protein
*SCO1*	19:30290001-30300000	7.01	0	0.26	To incorporate two Cytochrome c oxidase subunits
*ABCB10*	28:490001-500000	7.00	0.73	0.23	To participate in transports of molecules across cellular membrane
*NDUFAF6*	14:71630001-71640000	5.95	0.55	0.27	To regulate of biogenesis of subunit ND1 that is crucial for assembly of the mitochondrial respiratory chain complex I
*IMMP2L*	4:57730001-57740000	5.18	0	0.28	To process signal peptide sequences used to direct mitochondrial proteins to the mitochondria
*SPATA19*	29:33750001-33760000	8.36	0.077	0.23	Possibly to participate in spermiogenesis
*HSD3B1*	3:23810001-23820000	6.16	0	0.27	To catalyze the oxidative conversion of steroid precursors for the production of steroid hormones
*TRAK2*	2:90350001-90360000	31.40	0.49	0.3	Possibly to regulate endosome-to-lysosome trafficking of membrane cargo
*WWOX*	18:5720001-5730000	4.96	0.10	0.27	To act as a tumor suppressor and to induce apoptosis
*SLC30A6*	11:14780001-14790000	0	0.69	0.27	To regulate cytoplasmic level of zinc
*MYH6*	10:21350001-21360000	4.58	0	0.24	To participate in muscle contraction
*CASP8*	2:90290001-90300000	5.52	0.036	0.24	To induce in apoptosis
*FYN*	9:39140001-39150000	4.02	0.27	0.36	To control cell growth and survival as tyrosine kinase
*TMEM71*	14:9630001-9640000	6.12	0	0.24	Putative transmembrane protein
*PNPLA7*	11:105600001-105610000	15.13	0	0.36	To regulate adipocyte differentiation
*XAF1*	19:25750001-25760000	9.76	0.24	0.29	To regulate apoptosis by binds to the inhibitor of apoptosis protein family
*ZFHX3*	18:38410001-38420000	5.58	0	0.29	To regulate myogenic and neuronal differentiation.

For all N-mt windows, *F*_*st*_ between AFZ and EUT/AST and *F*_*st*_ between AFZ and ASZ were stratified based on the level of *F*_*st*_ between taurine (EUT/AST) and zebu (ASZ) (Fig. 5b). Our 29 mitonuclear selection candidate windows were enriched in the high differentiation quartiles (third and fourth quartiles) with Fisher’s exact test *P* value of 0.002, implying that the candidate selected loci were formerly distinct between unadmixed lineages of taurine and zebu. Additionally, our selection scan detected the regions distinct between AFZ and ASZ, 27 of 29 mitonuclear selection candidate windows were simultaneously located at the lower ends of *F*_*st*_ between AFZ and EUT/AST. This pattern contrasts our genomic analyses showing that AFZ are more differentiated from EUT/AST (genome-wide mean of *F*_*st*_ = 0.425) than from ASZ (genome-wide mean of *F*_*st*_ = 0.061). Therefore, these mitonuclear selection candidate windows trace back their ancestries to the taurine ancestors unlike the genome-wide trend of zebu-enriched ancestry.

Brief functional summary and Gene Ontology (GO) functional annotation of the 21 mitonuclear selection candidate genes are summarized in Table 3 and Additional file 3: Table S4, while significantly enriched GO terms (enrichment *P* value ≤0.05) being listed in Additional file 3: Table S5. As we selected N-mt genes based on MitoCarta 2.0 and GO:0005739, they were enriched in mitochondrial components as well as in biological processes related to the organization of mitochondrial respiratory chain complex. Among the high-confidence N-mt genes determined based on MitoCarta 2.0, *IMMP2L*, *SCO1*, and *NDUFAF6* have been reported to have a direct effect on cellular respiration (Additional file 3: Table S6). *IMMP2L*, inner mitochondrial membrane peptidase subunit 2, encodes a subunit which is necessary for the catalytic activity of the mitochondrial inner membrane peptidase. In mice, mutation of *IMMP2L* increased oxidative stress and leads to reduced fertility [61]. Reciprocal mtDNA replacements between allopatric mouse populations led to a reduction in fertility due to mitonuclear incompatibility, possibly due to *IMMP2L* allele incompatibility [62]. *SCO1*, synthesis of cytochrome C oxidase 1, encodes a protein contributing to the assembly of the key factor of cellular respiration, which transfers reducing equivalents to oxygen and pumps protons across the inner mitochondrial membrane. This gene is involved in mitochondrial complex IV deficiency, and mutation of this gene affects neonatal liver and brain development [63]. *NDUFAF6* encodes the ubiquinone oxidoreductase complex assembly factor 6 that plays a critical role in the respiratory chain complex I assembly. Therefore, its malfunction leads to mitochondrial respiratory chain complex I deficiency and regression of developments at an early age in humans [64–66]. These selection candidate genes showed similar patterns of taurine-enriched local ancestry in the genomes of African humped cattle, such as genotypes of the intragenic variants in the *NDUFAF6* (Fig. 5c, d, and Additional file 3: Fig. S8).

## Discussion

In this study, we first illustrate the ancestral characteristics of the admixed genome of African humped cattle using full mitochondrial and nuclear genomes. Our survey indicates (i) an absence of mitochondria of zebu origin, presumably due to its replacement with taurine mito-lineage T that entered Africa via Egypt (Fig. 1a and b), and (ii) a contrasting observation of major Asian zebu-originated ancestry in autosomes and *Y* chromosome that was introduced via the Horn of Africa (Figs. 1c, d and 2a, b) [8]. These results concur with previous reports of African humped cattle, where a breeding scheme of male-biased zebu introgression into African taurine was postulated to be a driver of this genomic discrepancy of African humped cattle [16–18, 23, 67]. Although this hypothesis based on male-biased zebu introgression seems to agree with the status of today’s African humped cattle genomes, it mandates the assumption that the mitochondrial heritage of female zebu in African humped cattle was initially absent or subsequently lost following random genetic drift. A notable prevalence and geographical influence of Asian zebu ancestry led us to suppose additional evolutionary forces to address the genome discrepancy in African humped cattle, the absence of zebu mitochondria co-existing with zebu-enriched genome.

In this study, we incorporated a hypothesis based on the compatibility between mitochondrial and N-mt genes during hybridization between isolated populations [28]. When two isolated populations are firstly admixed, N-mt and mitochondrial alleles of one population are exposed to distinct N-mt and mitochondrial alleles of the other population that they have not been co-evolving with. Thus, this co-introgression of mitochondrial haplotype and N-mt alleles would generate hybrid offspring with new combinations of N-mt alleles and mitochondrial haplotype of which genetic compatibility might not be reconciled and potentially cause the loss of fitness. Unless the introgressed materials are sufficiently beneficial to compensate for this fitness loss [37], mitonuclear incompatibility would exert selection pressure on the N-mt and mitochondrial loci. Then, the fitness loss would be reconciled by rapid displacement of N-mt alleles and mitochondria that account for the minority of the N-mt–mitochondria pool [37]. The direction of this mitonuclear selection depends on the prevalence of N-mt–mitochondrial pairs within the admixed population. Based on this hypothesis of mitonuclear selection, we assessed demographic scenarios that would have resulted in the current status of African humped cattle.

Accordingly, we hypothesized two selection pressures in the simulation, (i) male-biased zebu selection that is possibly induced by the pastoralists’ preference of large zebu bulls to taurine bulls, and (ii) mitonuclear selection that is driven by fitness loss in organisms carrying incompatible N-mt–mitochondrial pairs. We set *N*_*e*_ hyperparameters of our pulse-like admixture model to reflect the historical context of continuous zebu influxes into ancestral African cattle population over several centuries [8, 9]. Although the exact parameters of these zebu influxes are unknown, the estimation of historical *N*_*e*_ changes suggested that no significant population expansion has occurred since the time of admixture (Additional file 3: Fig. S3). That is, the demography of zebu × taurine admixture would have followed the form of zebu introgression into a large number of taurine cattle, rather than the form of zebu introgression into a small population followed by population expansion. Therefore, the admixture size *N*_*e*_ should be similar or larger than *N*_*e*_ of a single population of African humped cattle at 110 generations ago [14, 54].

By comparing the posterior probabilities of different models, the results of ABC suggest that cooperation of both selections explains the current genome discrepancy of African humped cattle better than other models implementing each of selection pressures alone (Table 2). The “zmsel” model assuming only male-biased zebu selection augments the zebu autosomal and *Y* chromosomal ancestry during admixture but requires a substantial size of zebu female population (*F*_*zf*_ ) to ensure the zebu-enriched ancestry (Fig. 4b and Additional file 3: Table S3). This sizeable zebu female population, however, hindered the “zmsel” model from reproducing the loss of zebu mitochondria during random genetic drift (Fig. 4a). Simultaneously, under the “mnsel” model assuming only mitonuclear selection, the demographic parameters pursuing the zebu-enriched genome ancestry also promotes zebu mitochondrial haplotype, thus not reproducing the loss of zebu mitochondria (Fig. 4a). Under the “bothsel” model includes both selection pressures, the preference of zebu bulls could increase the level of zebu genome ancestry, while mitonuclear selection during admixture simultaneously erases the minor fraction of zebu mitochondria.

Assuming the cooperation of both selection pressures, N-mt and mitochondrial loci of African humped cattle would have undergone co-adaptation to the mitonuclear selection pressure. We believe that the absence of zebu mitochondria represents the co-adaptative signature on mitochondrial loci, in a form of displacement of minor mitochondrial haplotype. To search for footprints of mitonuclear selection on N-mt loci, we scanned African cattle genomes and detected selection signatures at 21 N-mt gene loci. Simultaneously, local ancestries of African humped cattle were enriched with taurine alleles in these selection signatures (Fig. 5b), which contrasts their overall zebu-enriched genome-wide ancestry (Fig. 2a). Coupled with the absence of zebu mitochondria, this observation complies with the key expectation of the “bothsel” model, as shown in the accepted MN and MT values (Fig. 4a). It also corresponds to the co-introgression of N-mt–mitochondria between two *Drosophila* species followed by displacement of one mitochondrial haplotype [68]. Therefore, the prevalence of taurine genotypes in mitochondrial and N-mt loci in African humped cattle presumably have resulted from co-introgression of taurine mitochondria and N-mt alleles followed by mitonuclear selection.

We should note that the selection scans used in this study are known to be robust for detecting soft selective sweeps [57, 58]. As N-mt alleles have existed as standing variations in the unadmixed ancestral populations, selection from standing variations likely results in soft selective sweeps that are distinct from hard selective sweeps typically found in the case of selection from de novo variants [58, 69]. We also note that the heterozygosity shown in the present ASZ population (Fig. 5d) may reflect the recent admixture of ASZ with taurine cattle [70], which might have underestimated the signals of our cross-population selection scan between AFZ and ASZ.

The mitochondrial localization of these N-mt candidate gene products is supported by the experimental database of localization and expression of human N-mt genes in MitoCarta 2.0 (Additional file 3: Table S6) [60]. In particular, we identified *IMMP2L*, *NDUFAF6*, and *SCO1*, all of which are involved in the assembly of the mitochondrial respiratory chain complex. Given that these genes are crucial for cellular maintenance and function [61–66], the impairment of the compatibility at these N-mt loci may have caused the loss of fitness in hybrid cattle [30, 32]. As modes, targets, and level of mitonuclear incompatibility are complex and depend on the level of divergence between two populations at N-mt and mitochondrial loci [71], the exact mechanisms of mitonuclear incompatibility in taurine × zebu hybrids should be investigated further.

There have been a few experimental reports in the family *Bovidae* potentially supporting the onset of fitness loss from mitochondrial incompatibility in cattle hybridization. Bison introgressed with taurine mitochondria showed reduced growth compared to wild-type bison [72–74]. Cytoplasmic hybrids of taurine and yak (taurine cattle cell carrying yak mitochondria) have lower levels of viability compared to the normal taurine cattle cell due to impaired metabolisms [75]. It has also been documented in the 1970s that F2 of taurine × zebu hybrids showed lower fertilities compared to F2 of unadmixed taurine [76–78]. Although this reproductive depression in F2 of crossbred cattle may be linked to the reduced viability of germ cells [79–81], no definitive explanations have been suggested. Last but not least, a growing number of studies in a handful of mammalian species support a central role of mitonuclear incompatibility in genome integrity for hybridization and speciation [29, 62, 82].

## Conclusions

Although the nuclear genome of African humped cattle predominantly derives from zebu ancestors, we confirmed a contrasting observation of the absence of zebu mitochondrial ancestry. Male-biased zebu introgression was previously suggested to interpret this unique genome discrepancy; however, it heavily relies on extreme demographic assumptions or random genetic drift. This study is the first to apply the hypothesis based on selection induced by mitonuclear incompatibility to explain the absence of zebu mitochondrial ancestry. Indeed, our ABC result supports the cooperation of both male-biased zebu introgression and selection induced by mitonuclear incompatibility in the genome discrepancy of African humped cattle. Furthermore, we detected selection signatures at N-mt loci of the genome of African humped cattle that are also enriched with taurine ancestries. ABC and the supporting evidence indicate that the genome discrepancy in African humped cattle is likely shaped via the co-introgression of taurine mitochondrial and N-mt alleles followed by mitonuclear selection. Our findings provide a novel perspective on African cattle demography while further contributing to the research spectrum of the mitonuclear incompatibility hypothesis in hybrid populations.

## Methods

### Whole-genome re-sequencing

A total of 235 Illumina Hiseq 2000 WGS samples were used in the present study. In total, 121 samples—48 African cattle (Ankole, Kenana, Kenyan Boran, N’Dama and Ogaden) [14], 53 European/Asian *B. taurus* cattle (Angus, Jersey, Hanwoo and Holstein) [14, 83, 84], and 20 Asian *B. indicus* (Brahman, Gir, and Nelore) [85, 86]—were obtained from a public NCBI database. Additionally, 114 African cattle samples (Afar, Arsi, Barka, Butana, Ethiopian Boran, Fogera, Goffa, Horro, N’Dama, Mursi, and Sheko) were generated from a recent study of our research group [9] (Additional file 1: Table S1). Prior to multi-sample variant calling, per-base read qualities of all 235 WGS samples were checked using fastQC v11.4 [87]. After trimming raw reads using Trimmomatic v0.36, pair-end reads were mapped against the bovine reference genome UMD 3.1 using Bowtie v2.2.3 [39] using default parameters with “—no-mixed” to only align paired reads.

For autosomal variant calling of 235 WGS samples, we removed PCR duplicates with “REMOVE_DUPLICATES = true” using “MarkDuplicates” and fixed mate-pair using “FixMateInformation” in PicardTools v1.138 (http://broadinstitute.github.io/picard/). SAMtools v1.2 [88] was used to index the reference and alignment. “RealignerTargetCreator” and “IndelRealigner” of Genome analysis toolkit v3.4 (GATK) [89] were used to realign reads following misalignments caused by indels locally. “BaseRecalibrator” of GATK was used to recalibrate base qualities. “UnifiedGenotyper” and “SelectVariants” of GATK were used to call SNPs. “VariantFiltration” of GATK was used to filter out false-positive variants, with the following criteria: (i) SNP clusters evaluated with clusterSize of 3 and clusterWindowSize of 10; (ii) SNPs with a phred-scaled quality score less than 30 were filtered out; (iii) SNPs with MQ0 (mapping quality zero; total count across all samples of mapping quality zero reads) over 4 and quality depth less than 5 were filtered out; (iv) SNPs with FS (phred-scaled *P* value using Fisher’s exact test) over 200 were filtered out to avoid variation on either the forward or the reverse strand.

### *Y* chromosome sequence preparation

Here, we used the *Y* chromosome reference sequence of Btau4.6 assembly. After aligning WGS reads and removing PCR duplicates, we calculated depth coverage for each chromosome using Mosdepth v0.2.6 [90], only using reads with mapping quality over zero (Additional file 1: Table S1). We first calculated the depth ratios of the sex chromosomes as the average depths of *X* and *Y* chromosomes divided by the average depth of all autosomes. We then imputed the sex of the 235 WGS samples based on the Euclidean distance between the observed depth ratios and the expected depth ratio of autosomes: *X* chromosome: *Y* chromosome (1:0.5:0.5 for male and 1:1:0 for female). A total of 67 male samples were identified, and *Y* chromosomal variants from these samples were called based on the 1000 bull genome project pipeline [40]. First, we mapped WGS reads against using BWA [91]. We removed PCR duplicates using “MarkDuplicates” of PicardTools and corrected artifacts in base quality using “BaseRecalibrator” of GATK v3.8.1 with bqsrBAQGapOpenPenalty of 45. We generated individual GVCF for each sample using “HaplotypeCaller,” and we combined GVCFs to apply “SelectVariant” and “VariantFiltration” with the following options: (i) SNPs with a phred-scaled quality score < 30, (ii) SNPs with depth < 3, and (iii) Biallelic SNPs.

### Mitochondrial sequence preparation

A total of 494 mitochondrial sequences were analyzed in this study. We initially collected previously released complete mitochondrial genomes of *Bos* genus (“*B. taurus*,” “*B. indicus*,” and “*B. primigenius*”) and outgroup sequences (yak, “*Bos grunniens*” and bison, “*Bison bison*”) from the NCBI public database (National Center for Biotechnology Information. www.ncbi.nlm.nih.gov. Accessed 22 June 2019) using Entrez Direct [92]. Based on Genbank information [93], we then excluded (i) sequences without both “breed” and “country” information, and (ii) sequences shorter than 16,300 bps or longer than 16,500 bps. This resulted in 278 remaining reliable sequences for downstream analyses. Geographical origins were inferred by NCBI GenBank information or breed name, and furtherly clustered by subcontinental region (Standard Country or Area Codes for Statistical Use-M49 Standard, United Nations).

Additionally, we successfully assembled 216 mitochondrial sequences from publicly available WGS data using the method described in Qiu et al. (2015). To do so, we mapped WGS reads against the mitochondrial reference genome of ARS-UCD 1.2 (Accession no. NC_006853). We followed the same procedure as described in the *sample collection and re-sequencing* section, but we adjusted the last step of GATK “VariantFiltration” as follows: We excluded (i) SNPs and indels with a phred-scaled quality score < 30, (ii) SNPs with depth < 3, and (iii) Biallelic SNPs. Whole mitochondrial genome sequences were then generated by substituting missing SNPs or indels with the reference mitochondrial genome sequence. Following the manual curation, 19 Asian zebu mitochondrial sequences with mean value of read depth less than 10 and/or with flank region in alignment of more than two sequential variants were excluded. We remain then with a single Asian zebu sample I_ASZ_001@ (BioSample SAMN05788524). After removing these 19 ASZ mitochondrial sequences, 216 sequences remained for analysis (sequence materials are provided in Additional file 4: Dataset S1) and combined with the 278 reliable published sequences, which gives us a total of 494 mitochondrial DNA sequences for analysis.

### Maximum-likelihood phylogenetic reconstruction

To investigate the mitochondrial phylogeny, we performed multiple sequence alignment of the 494 mitochondrial sequences using MAFFT v7.397 [94]. A total of 16,338 nucleotide sites remained for analysis after removal of sites with a missing rate of 0.1 or more. Based on the annotation of the reference sequence, the aligned sequences were partitioned into the control region (D-loop), non-coding sequence (tRNA and rRNA regions), and coding sequence (genes regions). We then reconstructed a maximum-likelihood mitochondrial phylogeny using IQ-TREE v1.6.11 [95] with the following options: ModelFinder [96] with “-mset phyml” and “-msub mitochondrial”, and 1000 ultrafast bootstraps [97].

For genome-wide phylogenetic reconstruction, autosomal and *Y* chromosomal SNPs were filtered using PLINK v1.90 [98]. Autosomal SNPs with a minor allele frequency of 0.10 or higher (maf ≥ 0.10) and missing call rates of 0.01 or lower (missing ≤ 0.01) were filtered out, leaving 14,559,478 parsimony-informative SNPs for the phylogenetic analysis. *Y* chromosomal SNPs with a missing rate of 0.1 or more were filtered out, leaving 12,467 SNPs. Both autosomal and *Y* chromosomal SNPs were then used to reconstruct maximum-likelihood phylogenetic trees using IQ-TREE with a GTR model with ascertainment bias correction of the likelihood calculation (GTR + ASC) and 1000 ultrafast bootstraps. Additionally, we selected exonic autosomal variants to build a coding-region-based tree. A total of 125,671 exonic SNPs (maf ≥ 0.05) was extracted to reconstruct the coding-region-based phylogeny, using IQ-TREE with the same option as for the whole autosomal SNPs.

### Bayesian inference of evolutionary dynamics using mitochondrial sequences

Time points of the most recent ancestor were estimated using the BEAST v2.5.2 packages [99], with the following parameters: site model = GTR + G + I with empirical base frequencies, clock model = strict clock, tree model = coalescent constant population, chain length = 1 × 10^8^, burn-in = 1 × 10^7^, and a calibration of the common ancestry for *B. taurus*/*B. indicus* from bison/yak at 2 million years ago, according to the previous study [4]. BEAST results were assessed (ESS > 200) using Tracer v1.7.1, and the means for the time of the most recent ancestor for the haplogroups I, R, P, Q, T, and T1 were calculated using Treeannotator and marked on the maximum-likelihood tree. The discrete phylogeography was estimated for the 353 mitochondrial sequences from Africa and the adjacent regions (southern Europe, western Asia, and southern Asia) using BEAST and SpreaD3 v0.9.7 [100] with the non-redundant dispersals (Bayesian posterior probability ≥0.8) visualized.

### Population structure and demography estimation

Principal component analysis (PCA) and ancestry estimation were performed based on autosomal SNPs (maf ≥ 0.05 and missing rate ≤ 0.01) and *Y* chromosomal SNPs (missing rate ≤ 0.1), respectively. The covariance matrix of the top 20 principal components (PCs) was calculated using PLINK, and samples were projected on PC1 and PC2. To obtain a maximum-likelihood estimation of autosomal ancestry, we then implemented ADMIXTURE v1.3.0 [101] with the expected number of ancestry (*K*) of 2 and 3.

### Simulation of mitonuclear selection hypothesis

#### Admixture model

To test the mitonuclear seleion hypothesis, we performed ABC by simulating the admixture process using an in-house R script *abc_simulate.R* (github.com/TaehyungKwon/abc_simulation/). On the basis of the simulation model, we presumed two unadmixed ancestral populations, ancestral African taurine (denoted as “pre-T”) and ancestral Asian zebu immigrants (denoted as “pre-Z”). We modeled an admixture process to be a pulse-like admixture by fitness-based mating between pre-T and pre-Z (Fig. 3). The admixture generates post-admixture descendants (denoted as “post-A”) that correspond to the homogenous admixed population represented by AFZ. To apply realistic demography, we estimated the time of the admixture (generation *i* ago) using ALDER v1.03 [51] and the relative changes in historical *N*_*e*_ (*dN*) using SMC++ v1.15.3 [52] using autosomal SNPs of 101 AFZ samples, as implemented in function *in_popsize* of *abc_simulate.R*. For ALDER analysis, we used a minimum distance of 0.5 cM using SNPs with a missing rate of 0.01 or less and minor allele frequency of 0.01 or more. EUT/AST (*n* = 53) and ASZ (*n* = 20) were used as reference groups. For SMC++ analysis, we used “estimate” function of SMC++ with the following options: timepoint from generation 1 to 10,000, thinning constant of 400, and per generation mutation rate of 1 × 10^−8^. In both analyses, we calculated variance by applying leave-one-out jackknifing for each of 29 autosomes. For the size of admixture, three presets of *N*_*e*_ at generation 0 were set to test random genetic drift: *N*_*e*_ = 500, 2000, and 5000.

#### Attributes of individual

We modeled each individual of a population to be composed of six attributes: GB, MN, MT, Y, SEX, and FIT (function *simulate* of *abc_simulate.R*). To simplify multi-loci nature of genome ancestry, we designed a continuous attribute GB that corresponds to the ancestry of autosomal loci that is distinguishable between pre-T and pre-Z and evenly distributed across the autosomes. For each individual, two GBs were assigned for each of haploids: GB.1 and GB.2. GB for pure taurine alleles is set to 0 while GB for pure zebu alleles being set to 1. We presumed recombination rate of 1 centi-Morgan per Mb and sex-averaged genetic map length of 25 Morgan in cattle [102] to approximate the meiotic cross-over for multi-loci ancestry (function *in_meiosis_recomb* of *abc_simulate.R*). Therefore, we implemented 25 autosomes of 100 Mb length with one cross-over per generation per chromosome. As all individuals at generation 0 were unadmixed thus carry homozygous GB, GB of generation 1 would not be affected by meiotic cross-over (function *in_initialize* of *abc_simulate.R*). During meiosis from generation 1 to *i*, each of GB haploids in a zygote is randomly sampled from 25 × (*i* − 1) ancestry fragments of a parental germ cell (generation *i* − 1) with the probability of average GB of two haploids (GB.*μ*), which follows a binomial distribution *B*(number of trials = 25 × (*i* − 1), probability = GB.*μ*). As a result, each of GB haploids of the zygote can be respectively sampled from a normal distribution *N*(mean = GB.*μ*, variance = GB.*μ* × (1 − GB.*μ*) / {25 × (*i −* 1)}) (function *in_evolve* of *abc_simulate.R*).

We designed MN, a discrete attribute representing haplotype on N-mt loci that are inherited following the Mendelian inheritance (function *in_meiosis_haplo* of *abc_simulate.R*). Likewise, we designed MT and Y, discrete variables representing mitochondrial and *Y* haplotypes, respectively. These MN, MT, and Y haplotypes were set to 0 for taurine haplotype or 1 for zebu haplotype. An individual is composed of a pair of MN (MN.1 and MN.2), a single MT, and a single Y. We designed SEX representing sex of an individual given by random sampling based on the male frequency of the population (SEX = 0 for female; SEX = 1 for male). We designed FIT representing fitness of an individual to be used as a relative sampling probability in the parent sampling process.

#### Parameters and prior distributions

We parametrized three demographic parameters (*F*_*zm*_, *F*_*zf*_, and *MF*) and two selection pressure parameters (*S*_*zs*_ and *S*_*mn*_). We parametrized the proportion of pre-Z individuals in the male pool as *F*_*zm*_, the proportion of pre-Z individuals in the female pool as *F*_*zf*_, and the male frequency of post-A as *MF*. Two selection pressures account for the hypotheses of male-biased zebu selection (*S*_*zs*_) and mitonuclear selection (*S*_*mn*_), which affect FIT of an individual following the equation below:

$$ \mathrm{FIT}=\left(1-{S}_{mn}\times \left|\mathrm{MN}-\mathrm{MT}\right|\right)\times \left\{1+{S}_{zs}\times \mathrm{SEX}\times \left(\mathrm{GB}\times \mathrm{Y}\right)\right\} $$

Here, MN and GB indicate the average value of MN haploids and GB haploids of an individual, respectively. In brief, *S*_*mn*_ reduces fitness if the ancestry of N-mt loci and mitochondrial haplotype mismatch, and *S*_*zs*_ increases fitness if the individual is a male with zebu-enriched ancestry and zebu *Y* haplotype. Therefore, two selection pressures affect relative sampling probability of the individual in mating (function *in_evolve* of *abc_simulate.R*).

All parameters were randomly sampled from uniform prior distributions to avoid complications from the biased prior sampling (function *simulate* of *abc_simulate.R*; Table 1). *F*_*zf*_ was sampled from the prior distribution of *U*(0, 0.5) as we presume zebu female minority. *MF* was sampled from the prior distribution of *U*(0.05, 0.5) as the male frequency should be higher than that of the modern domestic herd with artificial insemination (0.05) and lower than that of random sex sampling assumed in the natural population. (0.5). *S*_*mn*_ was sampled from the prior distribution of *U*(0, 0.2) where an individual with a mismatch between homozygous MN and MT haplotype could incur a minimum of 80% fitness compared to an individual with matching MN and MT. *S*_*zs*_ was sampled from the prior distribution of *U*(0, 100) where purebred zebu male theoretically could have a maximum of 101 times fitness compared of purebred taurine male.

#### Approximate Bayesian computation

To infer the parameter values, we applied the rejection ABC framework [38] implemented in R package *abc* [53] using an in-house R script *abc_summarize.R*. At the end of each simulation, the mean values of GB, MT, and Y for each simulated population were collected as simulated summary statistics. For the observed summary statistics, we used the average autosomal, *Y* chromosomal, and mitochondrial ancestry of 101 AFZ samples that were estimated in our precedent analyses. Here, we compared the posterior probabilities of four models based on two selection pressures: a model without any selection pressures “neutral” (*S*_*mn*_ = 0 and *S*_*zs*_ = 0), a model simulating the mitonuclear selection “mnsel” (*S*_*zs*_ = 0), a model simulating the male-biased zebu selection “zmsel” (*S*_*mn*_ = 0), and a model simulating both selection pressures “bothsel.” Each model was simulated for 1 × 10^7^ replicates. Euclidean distances between the simulated sets and the observed summary statistics were standardized using median absolute deviation of the simulated summary statistics. We performed rejection ABC with tolerances of 0.01 and 0.001 that retains simulated sets of the smallest 1% and 0.1% distance, using “abc” function (function *run_abc* of *abc_summarize.R*). We performed cross-validation of model selection using “cv4postpr” for 20 random replicates and the model selection using “postpr” function (function *run_model_selection* of *abc_summarize.R*). We performed goodness-of-fit test using “gfit” function with the following options: statistics = mean, number of replicates = 1000 (function *run_goodness_of_fit* of *abc_summarize.R*).

### Scan of mitonuclear selection signatures

To detect mitonuclear selection signatures in African zebu, we scanned genome-wide selection signatures in AFZ using autosomal SNPs (maf ≥ 0.01). We used a combined approach that includes three scanning methods on non-overlapping 10-kb windows—Weir and Cockerham’s *F*_*st*_ [56], XP-CLR [57], and nSL [58]. VCFtools v0.015 [103] was used to calculate the *F*_*st*_ between ASZ and AFZ. Prior to the XP-CLR and nSL analysis, we phased SNPs using BEAGLE v4.1 [104]. We calculated the XP-CLR score and normalized between ASZ and AFZ with a maximum number of SNPs = 500 and a minimum number of SNPs = 10. Selscan v1.2.1 [105] was used to calculate nSL with no limit of nSL extension, and nSL scores were normalized using “–norm” as suggested by the developer [105].

We compared the top 1% results of all three methods and 215 *B. taurus* UMD 3.1.94 genes overlapping with 310 windows that were supported by at least two scan results. We merged 1576 one-to-one *B. taurus* orthologous genes to human N-mt genes in Human MitoCarta 2.0 (T_mito and T_possible mito) [60] and 1178 genes assigned to Gene Ontology term GO:0005739~mitochondrion. Putative N-mt functions were then determined for the 215 candidate genes based on the combined list of 1801 Ensembl genes (Ensembl UMD 3.194, accessed 25 January 2019) [59]. Subsequently, a total of 21 mitonuclear selection candidate genes were functionally annotated based using DAVID v6.8 [106] and PANTHER v14.1 [107] database. Significantly enriched GO terms (Enrichment *P* value ≤ 0.05) and GO terms for individual genes were listed [106].

For the gene *NDUFAF6*, we visualized genic variants (maf ≥ 0.05) in genotype, ancestry estimation, and maximum-likelihood phylogenetic tree. Individuals in genotype and ancestry estimation were hierarchically clustered within each classification group. Missense variants were annotated using SnpEff v4.3 [108] then identified using SnpSift v4.3 [109]. Ancestry estimation was conducted using ADMIXTURE program. RAxML-NG v0.9.0 [110] was used to reconstruct maximum-likelihood tree based on unphased genotype using the following parameters: substitution model = GTGTR4 + G, ascertainment bias correction = ASC_LEWIS, and bootstrapped 1000 times.

## Supplementary Information

**Additional file 1: Table S1.** Summary of genome re-sequencing using whole-genome sequencing data.

**Additional file 2: Table S2.** Summary of mitochondrial genome sequences.

**Additional file 3:** **Figure S1.** Maximum-likelihood tree based on autosomal coding sequence variants. **Figure S2.** PCA plot of 67 male samples based on Y chromosomal SNPs. **Figure S3.** Historical effective population size change of African zebu. **Figure S4.** Results of the cross-validation for model selection. **Figure S5.** Distances between the accepted simulated sets and the observed statistics under each model. **Figure S6.** Results of goodness-of-fit test. **Figure S7.** Correlations between three selection scans. **Figure S8.** Ancestry estimation and maximum likelihood phylogeny based on the genic variants of NDUFAF6. **Table S3.** Mean of posterior distribution for parameters. **Table S4.** Functional annotation of 21 candidates of mitonuclear selection signature. **Table S5.** Enriched Gene Ontology terms of 21 candidate genes of mitonuclear selection signature. **Table S6.** MitoCarta 2.0 summary of 21 candidate genes of mitonuclear selection signature

**Additional file 4: Dataset S1.** Bos taurus and Bos indicus cattle mitochondrial genome sequences generated from 216 whole-genome sequencing data.

## Data Availability

All data generated or analyzed during this study are included in this published article and supplementary information files. NCBI Sequence Read Archive Biosample IDs of whole-genome sequencing data are available in the Additional file 1: Table S1. NCBI accessions for publicly available mitochondrial sequences are included in Additional file 2: Table S2. Mitochondrial genome sequences newly generated in this article are available in Additional file 4: Dataset S1. The in-house python and R scripts for ABC simulation are available in the github repository (https://github.com/TaehyungKwon/abc_simulation).
