# Author Correction: Mitonuclear incompatibility as a hidden driver behind the genome ancestry of African admixed cattle

**DOI:** 10.1186/s12915-022-01265-8

**Published:** 2022-03-09

**Authors:** Taehyung Kwon, Kwondo Kim, Kelsey Caetano-Anolles, Samsun Sung, Seoae Cho, Choongwon Jeong, Olivier Hanotte, Heebal Kim

**Affiliations:** 1 Department of Agricultural Biotechnology and Research Institute of Agriculture and Life Sciences, Seoul National University, Seoul, South Korea; 2 eGnome, Inc., Seoul, South Korea; 3 Callout Biotech, Albuquerque, New Mexico United States; 4 School of Biological Sciences, Seoul National University, Seoul, South Korea; 5 School of Life Sciences, University of Nottingham, Nottingham, UK; 6 LiveGene, International Livestock Research Institute (ILRI), Addis Ababa, Ethiopia; 7 The Centre for Tropical Livestock Genetics and Health (CTLGH), The Roslin Institute, The University of Edinburgh, Edinburgh, UK; 8 Interdisciplinary Program in Bioinformatics, Seoul National University, Seoul, South Korea

**Correction to: BMC Biology 20, 1–20 (2021)**

**https://doi.org/10.1186/s12915-021-01206-x**

The original article contained minor errors in Figs. 1 and 3 which have both since been corrected.

