# Supplementary material for: Mitonuclear incompatibility as a hidden driver behind the genome ancestry of African admixed cattle
Source: BMC Biol. 2022 Jan 17;20:20. doi: 10.1186/s12915-021-01206-x (PMC8764764; doi:10.1186/s12915-021-01206-x)
Supplement: Supplementary file 3 — Additional file 3: Figure S1. Maximum-likelihood tree based on autosomal coding sequence variants. Figure S2. PCA plot of 67 male samples based on Y chromosomal SNPs. Figure S3. Historical effective population size change of African zebu. Figure S4. Results of the cross-validation for model selection. Figure S5. Distances between the accepted simulated sets and the observed statistics under each model. Figure S6. Results of goodness-of-fit test. Figure S7. Correlations between three selection scans. Figure S8. Ancestry estimation and maximum likelihood phylogeny based on the genic variants of NDUFAF6. Table S3. Mean of posterior distribution for parameters. Table S4. Functional annotation of 21 candidates of mitonuclear selection signature. Table S5. Enriched Gene Ontology terms of 21 candidate genes of mitonuclear selection signature. Table S6. MitoCarta 2.0 summary of 21 candidate genes of mitonuclear selection signature [file 12915_2021_1206_MOESM3_ESM.docx]

**Mitonuclear incompatibility as a hidden driver behind the genome ancestry of African admixed cattle**

Taehyung Kwon,^1^ Kwondo Kim,^1,2^ Kelsey Caetano-Anolles,^3^ Samsun Sung,^2^ Seoae Cho,^2^ Choongwon Jeong,^4^ Olivier Hanotte,^5,6,7*^, Heebal Kim^1,2,8*^

^1^ Department of Agricultural Biotechnology and Research Institute of Agriculture and Life Sciences, Seoul National University, Seoul, South Korea

^2^ eGnome, Inc, Seoul, South Korea

^3^ Callout Biotech, Albuquerque, New Mexico, United States

^4^ School of Biological Sciences, Seoul National University, Seoul, South Korea

^5^ School of Life Sciences, University of Nottingham, Nottingham, United Kingdom

^6^ LiveGene, International Livestock Research Institute (ILRI), Addis Ababa, Ethiopia

^7^ The Centre for Tropical Livestock Genetics and Health (CTLGH), The Roslin Institute, The University of Edinburgh, United Kingdom.

^8^ Interdisciplinary Program in Bioinformatics, Seoul National University, Seoul, South Korea

^*^Joint corresponding authors

Corresponding authors’ email addresses:

Olivier Hanotte: [olivier.hanotte@nottingham.ac.uk](mailto:olivier.hanotte@nottingham.ac.uk); o.hanotte@cgiar.org

Heebal Kim: [heebal@snu.ac.kr](mailto:heebal@snu.ac.kr)

**Supplementary Figures**

Fig. S1. Maximum-likelihood tree based on autosomal coding sequence variants

Fig. S2. PCA plot of 67 male samples based on Y chromosomal SNPs

Fig. S3. Historical effective population size change of African zebu

Fig. S4. Results of the cross-validation for model selection

Fig. S5. Distances between the accepted simulated sets and the observed statistics under each model

Fig. S6. Results of goodness-of-fit test

Fig. S7. Correlations between three selection scans

Fig. S8. Ancestry estimation and maximum likelihood phylogeny based on the genic variants of *NDUFAF6*

**Supplementary Tables**

Table S3. Mean of posterior distribution for parameters

Table S4. Functional annotation of 21 candidates of mitonuclear selection signature

Table S5. Enriched Gene Ontology terms of 21 candidate genes of mitonuclear selection signature

Table S6. MitoCarta 2.0 summary of 21 candidate genes of mitonuclear selection signature

Fig. S1. Maximum-likelihood tree based on autosomal coding sequence variants

The colored bar indicates the classification of each sample. Bootstrap values over 0.6 are not marked.


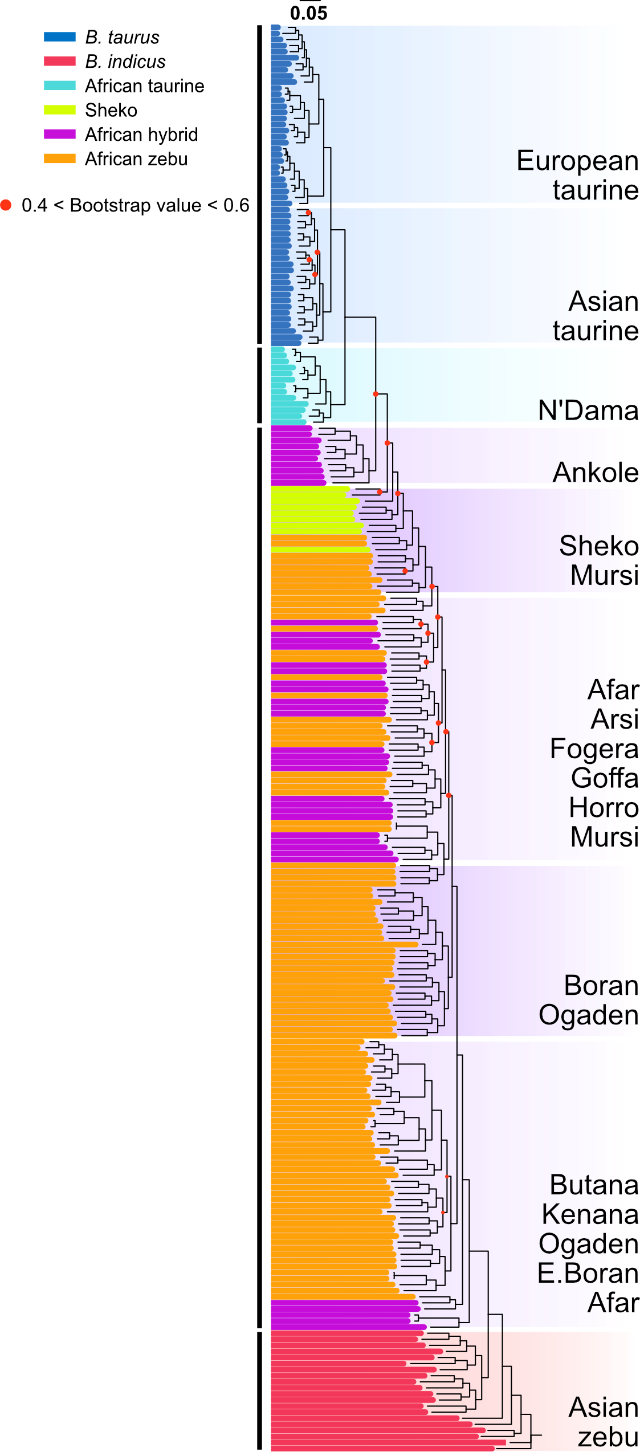


Fig. S2. PCA plot of 67 male samples based on *Y* chromosomal SNPs

**
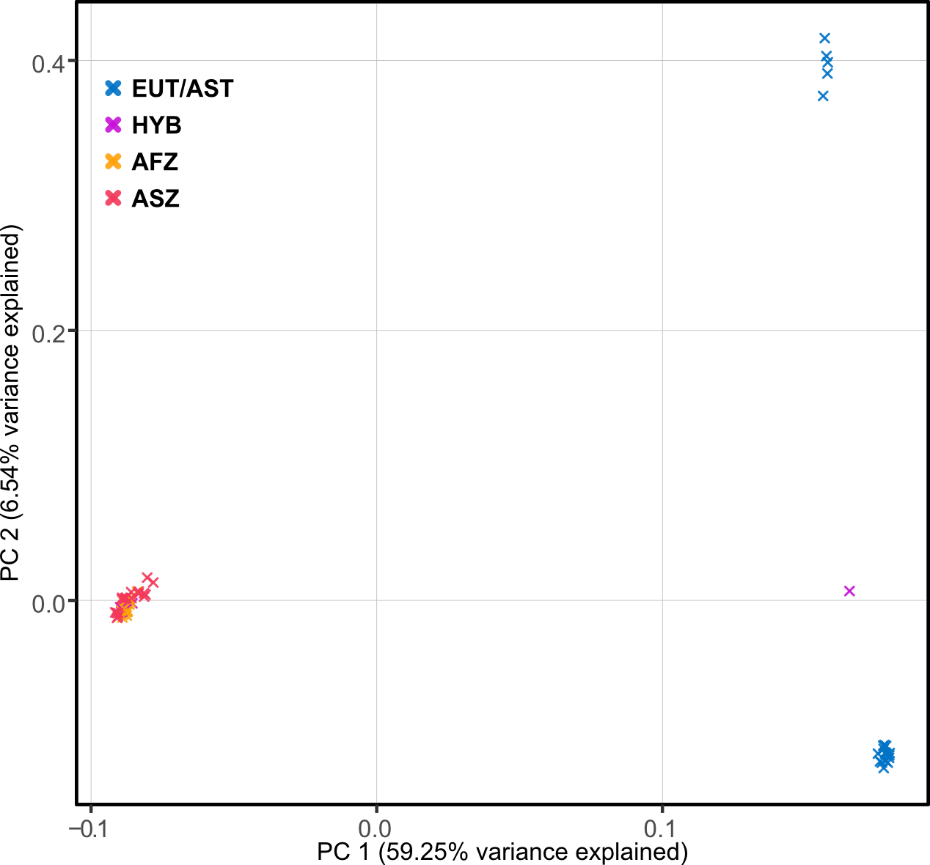
**

Fig. S3. Historical effective population size change of African zebu

Each red line indicates effective population size from each leave-one-out jackknifing set estimated using SNPs extracted in African zebu (n=101). Blue dashed line indicates the estimated time of admixture.

**
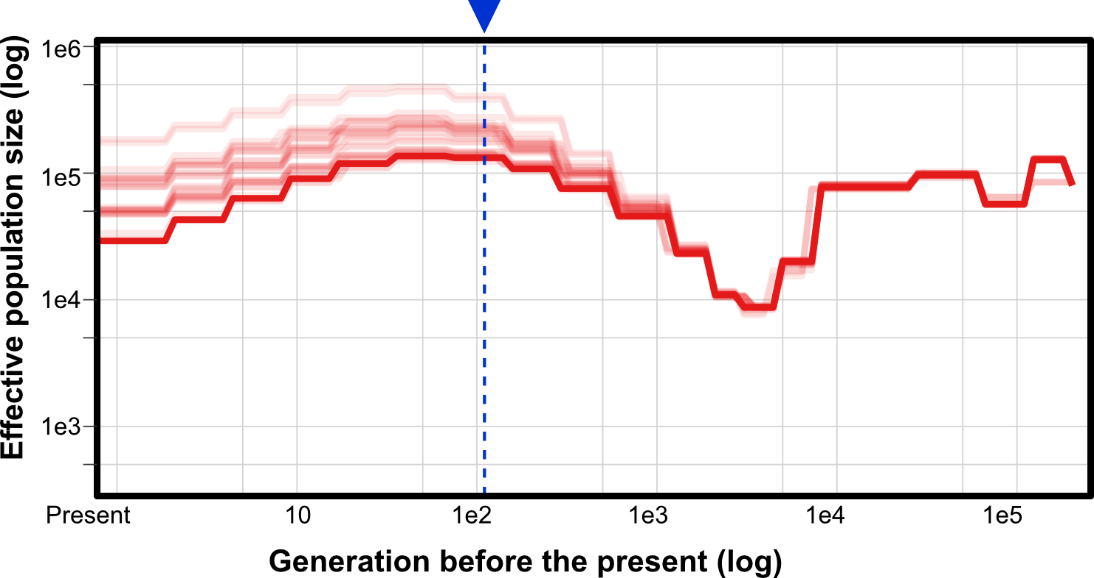
**

**Fig. S4. Results of the cross-validation for model selection**

Match between true model and predictive model is considered to be true positive. Each row indicates the proportion of true positives for each predicted model. Recall is calculated by sum of true positives divided by the total number of predictions.


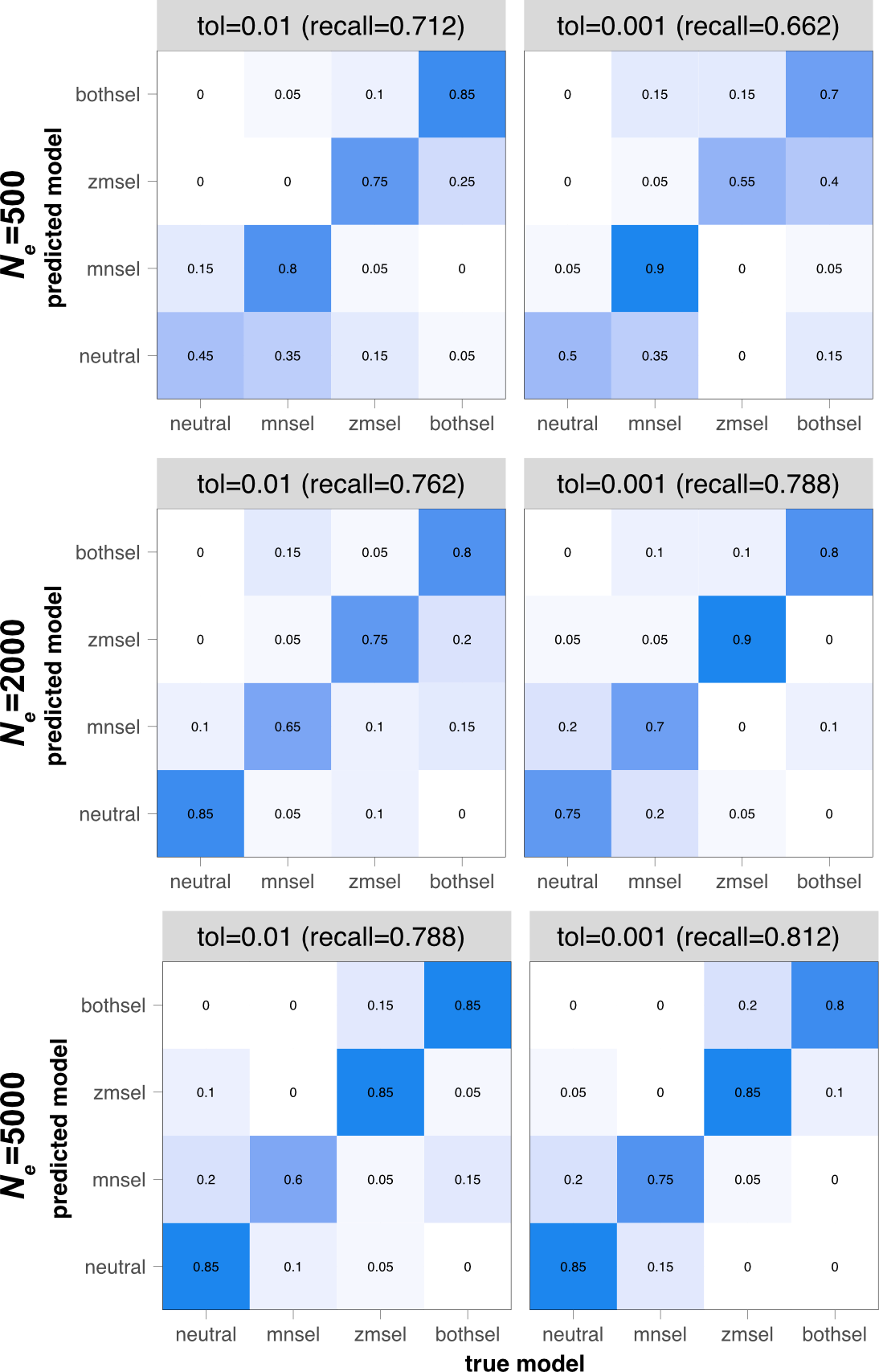


**Fig. S5. Distances between the accepted simulated sets and the observed statistics under each model**

**
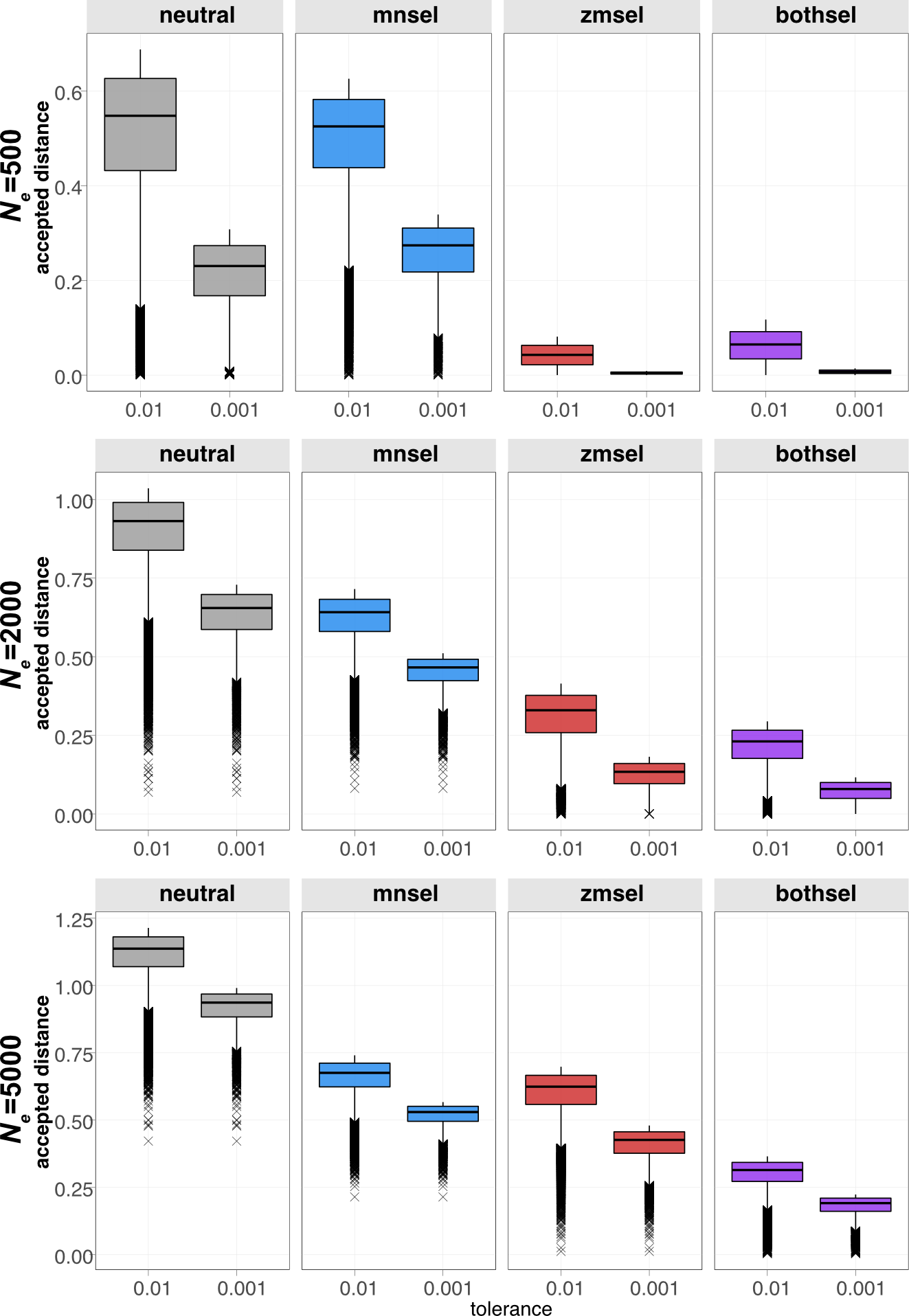
**

Fig. S6. Results of goodness-of-fit test

Null distributions of simulations under four simulation models were estimated from 100 simulation replicates. Blue line indicates the observed goodness-of-fit statistics calculated from the observed summary statistics. Red font indicates p-value under 0.05.


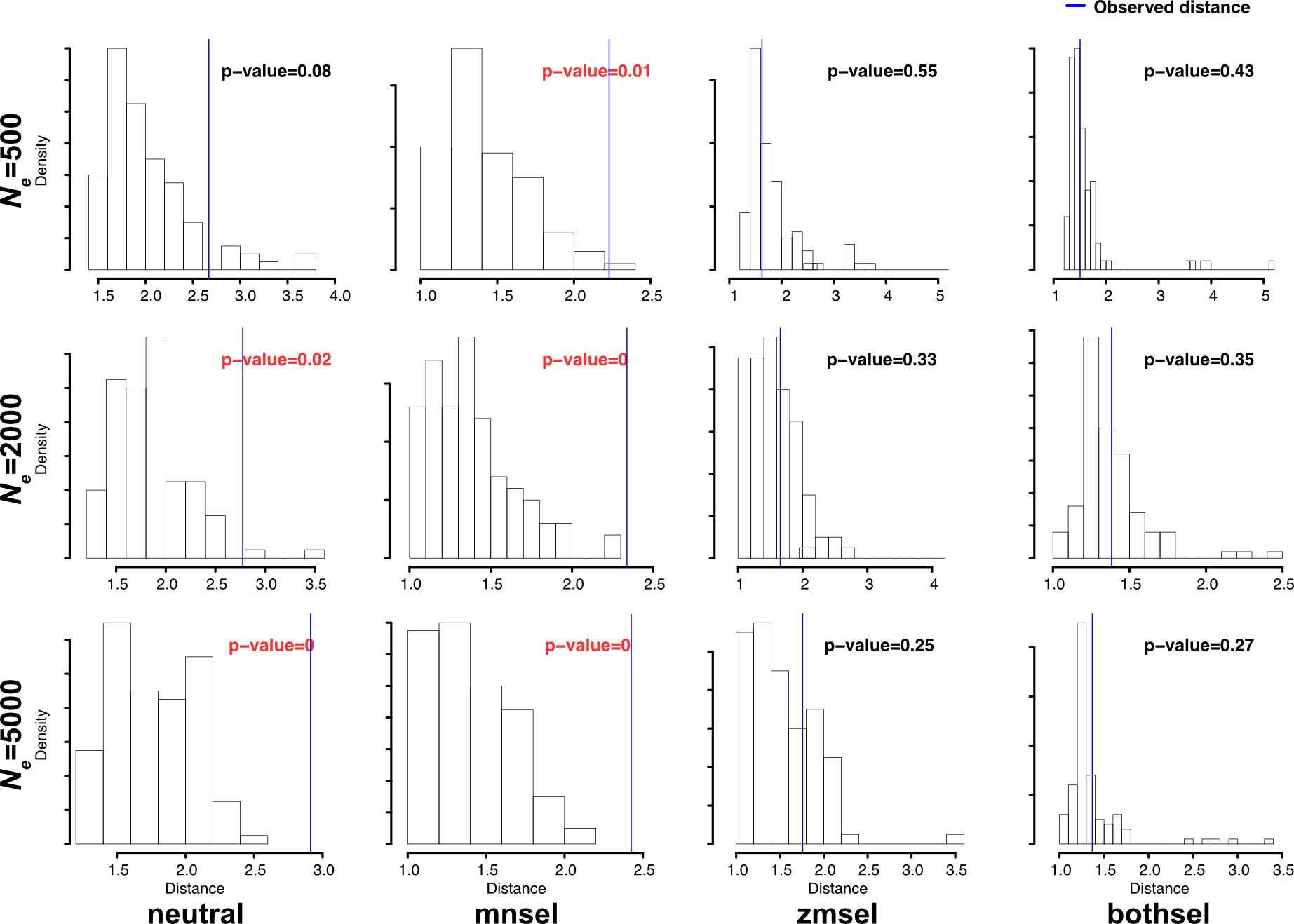


Fig. S7. Correlations between three selection scans

**
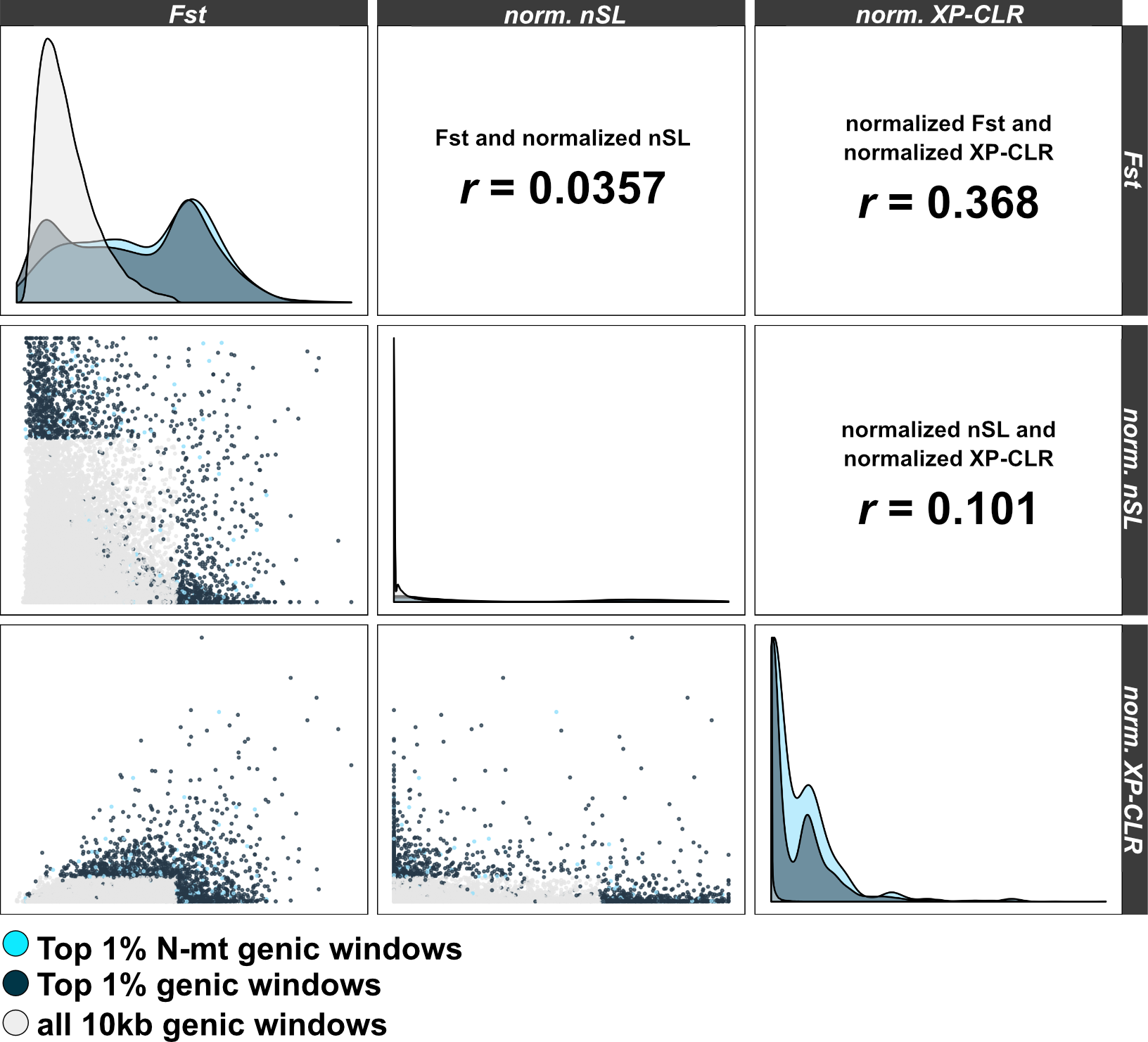
**

Fig. S8. Ancestry estimation and maximum likelihood phylogeny based on the genic variants of *NDUFAF6*

**
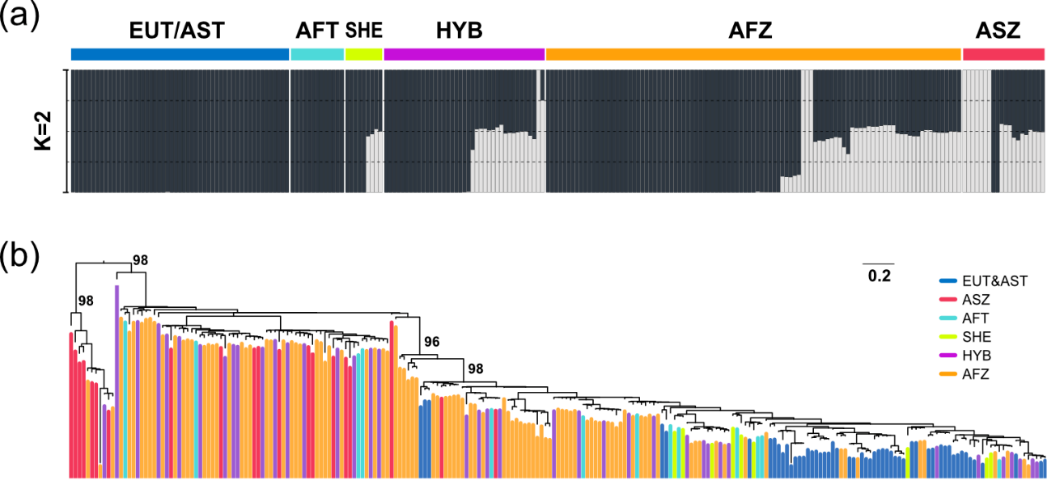
**

Table S3. Mean of posterior distribution for parameters

Numbers in the parentheses indicates 90% highest posterior density interval of the posterior distribution.

| ***N_e_*** | **model** | **tolerance** | ***F_zm_*** | ***F_zf_*** | ***MF*** | ***S_mn_*** | ***S_zs_*** |
| --- | --- | --- | --- | --- | --- | --- | --- |
| 500 | neutral | 0.01 | 0.931 (0.817-0.996) | 0.393 (0.27-0.49) | 0.29 (0.074-0.482) | - | - |
| 500 | neutral | 0.001 | 0.959 (0.889-0.998) | 0.446 (0.367-0.496) | 0.295 (0.071-0.485) | - | - |
| 500 | mnsel | 0.01 | 0.93 (0.814-0.996) | 0.372 (0.251-0.485) | 0.262 (0.066-0.477) | 0.077 (0.004-0.183) | - |
| 500 | mnsel | 0.001 | 0.951 (0.865-0.997) | 0.419 (0.32-0.492) | 0.246 (0.059-0.477) | 0.066 (0.002-0.174) | - |
| 500 | zmsel | 0.01 | 0.545 (0.102-0.957) | 0.317 (0.242-0.404) | 0.312 (0.087-0.486) | - | 54.544 (10.32-95.62) |
| 500 | zmsel | 0.001 | 0.54 (0.102-0.954) | 0.316 (0.244-0.401) | 0.315 (0.087-0.486) | - | 54.913 (10.45-95.45) |
| 500 | bothsel | 0.01 | 0.559 (0.116-0.958) | 0.285 (0.208-0.37) | 0.276 (0.068-0.481) | 0.1 (0.006-0.189) | 56.229 (12.28-95.86) |
| 500 | bothsel | 0.001 | 0.562 (0.115-0.959) | 0.286 (0.212-0.367) | 0.278 (0.068-0.481) | 0.108 (0.007-0.191) | 56.132 (12.73-95.87) |
| 2000 | neutral | 0.01 | 0.942 (0.842-0.997) | 0.307 (0.168-0.463) | 0.299 (0.077-0.483) | - | - |
| 2000 | neutral | 0.001 | 0.957 (0.881-0.997) | 0.373 (0.27-0.48) | 0.328 (0.092-0.488) | - | - |
| 2000 | mnsel | 0.01 | 0.948 (0.859-0.997) | 0.345 (0.238-0.48) | 0.268 (0.068-0.478) | 0.072 (0.003-0.184) | - |
| 2000 | mnsel | 0.001 | 0.964 (0.899-0.998) | 0.393 (0.29-0.489) | 0.259 (0.062-0.479) | 0.051 (0.001-0.174) | - |
| 2000 | zmsel | 0.01 | 0.57 (0.131-0.961) | 0.29 (0.224-0.372) | 0.324 (0.092-0.488) | - | 56.856 (13.16-96.02) |
| 2000 | zmsel | 0.001 | 0.582 (0.147-0.963) | 0.305 (0.258-0.358) | 0.356 (0.115-0.491) | - | 57.671 (13.7-96.05) |
| 2000 | bothsel | 0.01 | 0.587 (0.154-0.962) | 0.252 (0.203-0.319) | 0.269 (0.066-0.48) | 0.111 (0.004-0.191) | 58.934 (15.92-96.15) |
| 2000 | bothsel | 0.001 | 0.607 (0.173-0.966) | 0.266 (0.221-0.318) | 0.239 (0.058-0.477) | 0.125 (0.012-0.192) | 60.526 (17.459-96.32) |
| 5000 | neutral | 0.01 | 0.959 (0.886-0.998) | 0.217 (0.098-0.374) | 0.294 (0.077-0.483) | - | - |
| 5000 | neutral | 0.001 | 0.969 (0.911-0.998) | 0.275 (0.178-0.402) | 0.325 (0.088-0.488) | - | - |
| 5000 | mnsel | 0.01 | 0.957 (0.885-0.997) | 0.334 (0.233-0.479) | 0.271 (0.07-0.477) | 0.072 (0.002-0.185) | - |
| 5000 | mnsel | 0.001 | 0.971 (0.92-0.998) | 0.386 (0.275-0.49) | 0.268 (0.066-0.479) | 0.048 (0.001-0.179) | - |
| 5000 | zmsel | 0.01 | 0.588 (0.155-0.962) | 0.249 (0.186-0.33) | 0.321 (0.089-0.487) | - | 58.968 (15.62-96.17) |
| 5000 | zmsel | 0.001 | 0.602 (0.182-0.966) | 0.27 (0.22-0.333) | 0.358 (0.122-0.491) | - | 60.06 (17.288-96.18) |
| 5000 | bothsel | 0.01 | 0.602 (0.175-0.964) | 0.232 (0.198-0.288) | 0.269 (0.067-0.478) | 0.126 (0.004-0.193) | 60.765 (18.4-96.5) |
| 5000 | bothsel | 0.001 | 0.631 (0.211-0.97) | 0.243 (0.214-0.282) | 0.238 (0.058-0.478) | 0.137 (0.059-0.193) | 63.951 (22.31-97.08) |

Table S4. Functional annotation of 21 candidates of mitonuclear selection signature

| **Gene Symbol** | **GO Biological Process (Panther)** | **GO Biological Process (DAVID)** | **KEGG Pathway** |
| --- | --- | --- | --- |
| LYRM4 | - | - | - |
| COQ10A | - | - | - |
| TOMM40L | - | GO:0030150~protein import into mitochondrial matrix;GO:0055085~transmembrane transport | bta05014:Amyotrophic lateral sclerosis (ALS), |
| FARS2 | GO:0006432~phenylalanyl-tRNA aminoacylation | GO:0006432~phenylalanyl-tRNA aminoacylation;GO:0008033~tRNA processing | bta00970:Aminoacyl-tRNA biosynthesis, |
| MRPL41 | - | GO:0006412~translation | - |
| SCO1 | GO:0006825~copper ion transport;GO:0006878~cellular copper ion homeostasis;GO:0033617~mitochondrial respiratory chain complex IV assembly | GO:0006878~cellular copper ion homeostasis;GO:0033617~mitochondrial respiratory chain complex IV assembly;GO:1901799~negative regulation of proteasomal protein catabolic process | - |
| ABCB10 | GO:0034220~ion transmembrane transport | GO:0055085~transmembrane transport | bta02010:ABC transporters, |
| NDUFAF6 | GO:0032981~mitochondrial respiratory chain complex I assembly | GO:0009058~biosynthetic process;GO:0032981~mitochondrial respiratory chain complex I assembly;GO:0055085~transmembrane transport | - |
| IMMP2L | GO:0001541~ovarian follicle development;GO:0006465~signal peptide processing;GO:0006801~superoxide metabolic process;GO:0006974~cellular response to DNA damage stimulus;GO:0007283~spermatogenesis;GO:0007420~brain development;GO:0008015~blood circulation;GO:0022904~respiratory electron transport chain;GO:0030728~ovulation;GO:0061300~cerebellum vasculature development() | GO:0001541~ovarian follicle development;GO:0006465~signal peptide processing;GO:0006627~protein processing involved in protein targeting to mitochondrion;GO:0006801~superoxide metabolic process;GO:0006974~cellular response to DNA damage stimulus;GO:0007283~spermatogenesis;GO:0007420~brain development;GO:0008015~blood circulation;GO:0022904~respiratory electron transport chain;GO:0030728~ovulation;GO:0033108~mitochondrial respiratory chain complex assembly;GO:0061300~cerebellum vasculature development | bta03060:Protein export, |
| SPATA19 | GO:0007275~multicellular organism development;GO:0007283~spermatogenesis;GO:0030154cell differentiation | GO:0007275~multicellular organism development;GO:0007283~spermatogenesis;GO:0030154~cell differentiation | - |
| HSD3B1 | - | - | - |
| TRAK2 | - | - | bta01100:Metabolic pathways,bta04727:GABAergic synapse, |
| WWOX | - | GO:0001649~osteoblast differentiation;GO:0030178~negative regulation of Wnt signaling pathway;GO:0048705~skeletal system morphogenesis;GO:0071560~cellular response to transforming growth factor beta stimulus;GO:0072332~intrinsic apoptotic signaling pathway by p53 class mediator;GO:0097191~extrinsic apoptotic signaling pathway;GO:2001241~positive regulation of extrinsic apoptotic signaling pathway in absence of ligand | - |
| SLC30A6 | zinc ion transport(GO:0006829);cation transmembrane transport(GO:0098655) | GO:0010043~response to zinc ion;GO:0061088~regulation of sequestering of zinc ion;GO:0071577~zinc II ion transmembrane transport | - |
| MYH6 | - | GO:0001701~in utero embryonic development;GO:0002026~regulation of the force of heart contraction;GO:0002027~regulation of heart rate;GO:0007512~adult heart development;GO:0007522~visceral muscle development;GO:0008217~regulation of blood pressure;GO:0014898~cardiac muscle hypertrophy in response to stress;GO:0030049~muscle filament sliding;GO:0030509~BMP signaling pathway;GO:0043462~regulation of ATPase activity;GO:0045214~sarcomere organization;GO:0046034~ATP metabolic process;GO:0048739~cardiac muscle fiber development;GO:0055009~atrial cardiac muscle tissue morphogenesis;GO:0055010~ventricular cardiac muscle tissue morphogenesis;GO:0060048~cardiac muscle contraction;GO:0060070~canonical Wnt signaling pathway;GO:0060420~regulation of heart growth | bta04260:Cardiac muscle contraction,bta04261:Adrenergic signaling in cardiomyocytes,bta05416:Viral myocarditis, |
| CASP8 | GO:0006508~proteolysis;GO:0006915~apoptotic process;GO:0097194~execution phase of apoptosis() | GO:0006915~apoptotic process;GO:0008625~extrinsic apoptotic signaling pathway via death domain receptors;GO:0030225~macrophage differentiation;GO:0034612~response to tumor necrosis factor;GO:0036462~TRAIL-activated apoptotic signaling pathway;GO:0042981~regulation of apoptotic process;GO:0043123~positive regulation of I-kappaB kinase/NF-kappaB signaling;GO:0043124~negative regulation of I-kappaB kinase/NF-kappaB signaling;GO:0045651~positive regulation of macrophage differentiation;GO:0051603~proteolysis involved in cellular protein catabolic process;GO:0071260~cellular response to mechanical stimulus;GO:0097194~execution phase of apoptosis;GO:0097202~activation of cysteine-type endopeptidase activity | bta04115:p53 signaling pathway,bta04210:Apoptosis,bta04620:Toll-like receptor signaling pathway,bta04621:NOD-like receptor signaling pathway,bta04622:RIG-I-like receptor signaling pathway,bta04668:TNF signaling pathway,bta04932:Non-alcoholic fatty liver disease (NAFLD),bta05010:Alzheimer's disease,bta05016:Huntington's disease,bta05134:Legionellosis,bta05142:Chagas disease (American trypanosomiasis),bta05145:Toxoplasmosis,bta05152:Tuberculosis,bta05161:Hepatitis B,bta05168:Herpes simplex infection,bta05200:Pathways in cancer,bta05203:Viral carcinogenesis,bta05416:Viral myocarditis, |
| FYN | GO:0001764~neuron migration;GO:0002250~adaptive immune response;GO:0003015~heart process;GO:0008360~regulation of cell shape;GO:0010629~negative regulation of gene expression;GO:0010976~positive regulation of neuron projection development;GO:0030900~forebrain development;GO:0031397~negative regulation of protein ubiquitination;GO:0036120~cellular response to platelet-derived growth factor stimulus;GO:0042177~negative regulation of protein catabolic process;GO:0042531~positive regulation of tyrosine phosphorylation of STAT protein;GO:0043123~positive regulation of I-kappaB kinase/NF-kappaB signaling;GO:0045471~response to ethanol;GO:0048813~dendrite morphogenesis;GO:0050798~activated T cell proliferation;GO:0050804~modulation of chemical synaptic transmission;GO:0050966~detection of mechanical stimulus involved in sensory perception of pain;GO:0071560~cellular response to transforming growth factor beta stimulus;GO:0090314~positive regulation of protein targeting to membrane;GO:1900182~positive regulation of protein localization to nucleus;GO:1900449~regulation of glutamate receptor signaling pathway;GO:1901216~positive regulation of neuron death;GO:1902951~negative regulation of dendritic spine maintenance;GO:1904646~cellular response to amyloid-beta;GO:1905664~regulation of calcium ion import across plasma membrane() | GO:0001764~neuron migration;GO:0002250~adaptive immune response;GO:0007169~transmembrane receptor protein tyrosine kinase signaling pathway;GO:0007417~central nervous system development;GO:0008360~regulation of cell shape;GO:0010629~negative regulation of gene expression;GO:0010976~positive regulation of neuron projection development;GO:0016477~cell migration;GO:0030154~cell differentiation;GO:0030900~forebrain development;GO:0031397~negative regulation of protein ubiquitination;GO:0036120~cellular response to platelet-derived growth factor stimulus;GO:0038083~peptidyl-tyrosine autophosphorylation;GO:0042110~T cell activation;GO:0042127~regulation of cell proliferation;GO:0042177~negative regulation of protein catabolic process;GO:0042523~positive regulation of tyrosine phosphorylation of Stat5 protein;GO:0042981~regulation of apoptotic process;GO:0043123~positive regulation of I-kappaB kinase/NF-kappaB signaling;GO:0045087~innate immune response;GO:0045471~response to ethanol;GO:0048813~dendrite morphogenesis;GO:0050798~activated T cell proliferation;GO:0050852~T cell receptor signaling pathway;GO:0050966~detection of mechanical stimulus involved in sensory perception of pain;GO:0071560~cellular response to transforming growth factor beta stimulus;GO:1900182~positive regulation of protein localization to nucleus | bta04071:Sphingolipid signaling pathway,bta04360:Axon guidance,bta04380:Osteoclast differentiation,bta04510:Focal adhesion,bta04520:Adherens junction,bta04611:Platelet activation,bta04650:Natural killer cell mediated cytotoxicity,bta04660:T cell receptor signaling pathway,bta04664:Fc epsilon RI signaling pathway,bta04725:Cholinergic synapse,bta05020:Prion diseases,bta05162:Measles,bta05416:Viral myocarditis, |
| TMEM71 | - | - | - |
| PNPLA7 | GO:0016042~lipid catabolic process | GO:0006629~lipid metabolic process;GO:0032502~developmental process | bta00564:Glycerophospholipid metabolism, |
| XAF1 | GO:0006915~apoptotic process | GO:0006915~apoptotic process;GO:0035456~response to interferon-beta | - |
| ZFHX3 | GO:0071559~response to transforming growth factor beta | GO:0000122~negative regulation of transcription from RNA polymerase II promoter;GO:0045893~positive regulation of transcription; DNA-templated;GO:0071559~response to transforming growth factor beta | bta04550:Signaling pathways regulating pluripotency of stem cells, |

Table S5. Enriched Gene Ontology terms of 21 candidate genes of mitonuclear selection signature

Terms with enrichment *P*-value less than 0.05 are shown.

| **Category** | **Term** | **Count** | **P-value** | **Genes** |
| --- | --- | --- | --- | --- |
| GOTERM_BP_FAT | GO:0033108~mitochondrial respiratory chain complex assembly | 3 | 8.51E-04 | IMMP2L, NDUFAF6, SCO1 |
| GOTERM_BP_FAT | GO:0070848~response to growth factor | 4 | 0.010906757 | FYN, MYH6, ZFHX3, WWOX |
| GOTERM_BP_FAT | GO:0071559~response to transforming growth factor beta | 3 | 0.011741459 | FYN, ZFHX3, WWOX |
| GOTERM_BP_FAT | GO:0007005~mitochondrion organization | 4 | 0.027974451 | IMMP2L, NDUFAF6, TOMM40L, SCO1 |
| GOTERM_BP_FAT | GO:0010033~response to organic substance | 6 | 0.039166496 | FYN, CASP8, XAF1, MYH6, ZFHX3, WWOX |
| GOTERM_CC_FAT | GO:0005739~mitochondrion | 13 | 3.61E-08 | IMMP2L, NDUFAF6, MRPL41, HSD3B1, FARS2, LYRM4, CASP8, SPATA19, TOMM40L, ABCB10, XAF1, WWOX, SCO1 |
| GOTERM_CC_FAT | GO:0044429~mitochondrial part | 7 | 3.32E-04 | IMMP2L, NDUFAF6, MRPL41, HSD3B1, SPATA19, TOMM40L, ABCB10 |
| GOTERM_CC_FAT | GO:0031966~mitochondrial membrane | 6 | 8.07E-04 | IMMP2L, NDUFAF6, HSD3B1, SPATA19, TOMM40L, ABCB10 |
| GOTERM_CC_FAT | GO:0005740~mitochondrial envelope | 6 | 0.001053706 | IMMP2L, NDUFAF6, HSD3B1, SPATA19, TOMM40L, ABCB10 |
| GOTERM_CC_FAT | GO:0031967~organelle envelope | 6 | 0.006325363 | IMMP2L, NDUFAF6, HSD3B1, SPATA19, TOMM40L, ABCB10 |
| GOTERM_CC_FAT | GO:0031975~envelope | 6 | 0.006421639 | IMMP2L, NDUFAF6, HSD3B1, SPATA19, TOMM40L, ABCB10 |
| GOTERM_CC_FAT | GO:0044455~mitochondrial membrane part | 3 | 0.027982414 | IMMP2L, TOMM40L, ABCB10 |

Table S6. MitoCarta 2.0 summary of 21 candidate genes of mitonuclear selection signature

“MC” stands for MitoCarta 2.0 (Calvo et al., 2015). MSMS Score indicates MS/MS abundance (coverage) and enrichment in subtractive proteomics. MC score indicates sum of log-likelihood ratios for scoring TargetP, MitoDomain, CoexpressionGnfN50, PGC Induction, YeastMitoHomolog, RickettsiaHomolog, and MSMS). MC FDR indicates estimated corrected false discovery rate. MC evidence indicates types of experimental support.

| **Gene symbol** | **MC support** | **MSMS Score** | **MC score** | **MC FDR** | **MC evidence** | **Expression Tissues** |
| --- | --- | --- | --- | --- | --- | --- |
| LYRM4 | Tmito | 75-100pure | 29.009 | 0 | literature, APEX_matrix, targetP signal+, yeast mito homolog, mito protein domain+, induction, coexpression, MS/MS++ | all 14 |
| COQ10A | Tpossible_mito | 50-75pure | 28.7285 | 0 | targetP signal, yeast mito homolog+, Rickettsial homolog, mito protein domain+, induction, MS/MS++ | cerebrum, cerebellum, brainstem, spinalcord, kidney, liver, heart, skeletalmuscle, adipose, largeintestine, stomach |
| TOMM40L | Tpossible_mito | 25-50ambig | 25.4413 | 0 | targetP signal, yeast mito homolog+, mito protein domain+, induction, coexpression+, MS/MS | cerebrum, cerebellum, brainstem, spinalcord, adipose, largeintestine, stomach, testis |
| FARS2 | Tmito | 25-50ambig | 23.8332 | 0 | literature, APEX_matrix, targetP signal, yeast mito homolog+, Rickettsial homolog, mito protein domain, induction, coexpression, MS/MS | adipose, largeintestine, stomach, placenta, testis |
| MRPL41 | Tmito | 50-75pure | 19.7869 | 0 | literature, APEX_matrix, yeast mito homolog++, mito protein domain, induction, MS/MS++ | all 14 |
| SCO1 | Tmito | 50-75pure | 19.7152 | 0 | literature, APEX_IMS, APEX_matrix, targetP signal, yeast mito homolog+, Rickettsial homolog, mito protein domain, MS/MS++ | cerebrum, cerebellum, brainstem, spinalcord, liver, heart, adipose, smallintestine, largeintestine, stomach, placenta, testis |
| ABCB10 | Tmito | 25-50pure | 18.2703 | 0 | literature, APEX_matrix, yeast mito homolog+, Rickettsial homolog, mito protein domain, induction, coexpression, MS/MS+ | cerebellum, spinalcord, liver, heart, skeletalmuscle, adipose, smallintestine, largeintestine, stomach, placenta, testis |
| NDUFAF6 | Tmito | 25-50ambig | 16.9393 | 0 | literature, targetP signal, mito protein domain, induction, coexpression++, MS/MS | adipose, smallintestine, largeintestine, placenta, testis |
| IMMP2L | Tmito | 50-75ambig | 13.8041 | 0.004 | literature, yeast mito homolog++, mito protein domain, coexpression, MS/MS | cerebellum, brainstem, liver, skeletalmuscle, adipose, largeintestine, testis |
| SPATA19 | Tpossible_mito | 50-75pure | 9.7131 | 0.012 | mito protein domain+, MS/MS++ | testis |
| HSD3B1 | Tpossible_mito | 25-50pure | 4.5019 | 0.073 | 0 | kidney, liver, adipose, largeintestine, placenta, testis |
| TRAK2 | Tpossible_mito | 0-25ambig | 4.1433 | 0.094 | 0 | adipose |
| WWOX | Tpossible_mito | 0 | 3.7304 | 0.105 | 0 | 0 |
| SLC30A6 | Tmito | 0 | -0.9827 | 0.42 | GFP, targetP signal, mito protein domain | 0 |
| MYH6 | Tpossible_mito | 25-50crude | -3.4637 | 0.627 | 0 | cerebrum, brainstem, spinalcord, kidney, liver, heart, skeletalmuscle, adipose, smallintestine, largeintestine, stomach, placenta, testis |
| CASP8 | Tmito | 0 | -3.7952 | 0.639 | literature, mito protein domain | 0 |
| FYN | Tpossible_mito | 0 | -5.5448 | 0.739 | 0 | 0 |
| TMEM71 | - | 0 | -6.0415 | 0.762 | 0 | 0 |
| PNPLA7 | Tpossible_mito | 0 | -6.8878 | 0.827 | 0 | 0 |
| XAF1 | Tpossible_mito | 0 | -8.0704 | 0.845 | 0 | 0 |
| ZFHX3 | Tpossible_mito | 0-25ambig | -8.2811 | 0.869 | 0 | cerebrum, brainstem, stomach |
